# The tardigrade-derived mitochondrial abundant heat soluble protein improves adipose-derived stem cell survival against representative stressors

**DOI:** 10.1038/s41598-024-62693-w

**Published:** 2024-05-23

**Authors:** Jordan L. Rolsma, William Darch, Nicholas C. Higgins, Joshua T. Morgan

**Affiliations:** Department of Bioengineering, University of California, 900 University Ave, Riverside, CA 92521 USA

**Keywords:** Tardigrade, Stem cells, Transgenic, Stress tolerance, Injections, Cell biology, Cell death, Stem-cell research

## Abstract

Human adipose-derived stem cell (ASC) grafts have emerged as a powerful tool in regenerative medicine. However, ASC therapeutic potential is hindered by stressors throughout their use. Here we demonstrate the transgenic expression of the tardigrade-derived mitochondrial abundant heat soluble (MAHS) protein for improved ASC resistance to metabolic, mitochondrial, and injection shear stress. In vitro, MAHS-expressing ASCs demonstrate up to 61% increased cell survival following 72 h of incubation in phosphate buffered saline containing 20% media. Following up to 3.5% DMSO exposure for up to 72 h, a 14–49% increase in MAHS-expressing ASC survival was observed. Further, MAHS expression in ASCs is associated with up to 39% improved cell viability following injection through clinically relevant 27-, 32-, and 34-gauge needles. Our results reveal that MAHS expression in ASCs supports survival in response to a variety of common stressors associated with regenerative therapies, thereby motivating further investigation into MAHS as an agent for stem cell stress resistance. However, differentiation capacity in MAHS-expressing ASCs appears to be skewed in favor of osteogenesis over adipogenesis. Specifically, activity of the early bone formation marker alkaline phosphatase is increased by 74% in MAHS-expressing ASCs following 14 days in osteogenic media. Conversely, positive area of the neutral lipid droplet marker BODIPY is decreased by up to 10% in MAHS-transgenic ASCs following 14 days in adipogenic media. Interestingly, media supplementation with up to 40 mM glucose is sufficient to restore adipogenic differentiation within 14 days, prompting further analysis of mechanisms underlying interference between MAHS and differentiation processes.

## Introduction

Stem-cell-based therapies are increasingly prevalent^1^. As a result of the rapid expansion of research and clinical trials in this field, the global market for stem-cell-based therapies is projected to increase by 13.73% between 2022 and 2030 to a total value of $31.41 billion USD^2^. Human adipose-derived stem cells (ASCs) are a subset of mesenchymal stem cells that are frequently exploited as therapeutic agents due to their ease of isolation from adipose tissue, immunomodulatory effects, wound repair mediation, and multilineage differentiation capacity^3^. ASCs are isolated during minimally invasive, elective liposuction procedures, and their therapeutic use avoids the ethical controversy of embryonic stem cell extraction^4^. ASCs isolated from processed lipoaspirate secrete potent anti-inflammatory cytokines to suppress immune responses and paracrine factors to promote regeneration at wound sites, and possess multilineage differentiation potential for the regeneration of vasculature, muscle, tendon, cartilage, bone, adipose, and cardiac tissues^3,5–10^. For these reasons, ASCs are commonly used for the clinical regeneration of a wide range of diseased, damaged, avascular, and aged tissues^11–16^.

Despite this restorative potential, multiple factors negatively affect the efficacy of cellular therapies^17^. Cryopreservation of ASCs is commonly used for their long-term storage and transportation to treatment facilities. Conventionally used slow cooling causes cell dehydration and hyperosmotic stress due to solute concentration and solvent freezing^18,19^. This is mitigated in part through the use of cryoprotectants, such as dimethyl sulfoxide (DMSO). However, DMSO is associated with both loss of cell viability and clinical adverse effects. DMSO damages cells through several mechanisms, most notably through impairing membrane function. In mitochondria this can lead to loss of membrane potential and reactive oxygen species (ROS) generation, in the endoplasmic reticulum (ER) this can lead to calcium release and consequent induction of apoptosis^20–24^. Further, numerous clinical trials report DMSO-specific adverse effects in patients receiving stem cell therapies, including seizures, cardiac hypertension, and severe allergic reactions^25–30^.

While there are numerous treatment methods, cellular engraftment through injection is common. During injection, cell viability is known to decrease due to shear stress: direct impacts include membrane damage, cell lysis, morphology changes, and apoptosis^31–35^. Further, patient comfort and sensitive tissues (such as eyes, hearts, and joints) often motivate the use of higher needle gauges (i.e., of smaller diameter) to avoid damage and leakage along the needle tract^31,36–38^. Unfortunately, higher gauges impart more mechanical damage; one prior study showing viable retention as low as 1–32% 24 h to 7 days after controlled injection into wound sites^39–41^. Following injection or engraftment, cellular therapies can face additional metabolic stress resulting from nutrient-poor, ischemic, aged, injured, and otherwise impaired environments^42,43^. Cell starvation and hypoxia trigger ER stress and ROS production, resulting in the initiation of apoptotic signaling events^44^. These factors contribute to decreased cell life span and reduced therapeutic efficacy—multiple studies describe low MSC survival and retention after implantation into ischemic tissue (as low as 1–10% at 7 days) when dosed at 5000–50,000 cells/mL^32,45–51^. Overall, multiple stress factors reduce therapeutic cell viability and limit treatment efficacy. The stress incurred throughout the therapeutic cell lifecycle motivates the development of multi-stress tolerance for more cost-effective, patient-friendly stem cell therapies.

Transgenic protein expression is a useful tactic for conferring stress-resistant properties to mammalian cells. Indeed, transgenic expression of human glutathione reductase, peroxiredoxin 5, and MnSOD increases Chinese hamster ovary (CHO) cell resistance to oxidative stress^52–54^. Strikingly, human glutathione reductase expression reduces LDH media concentration in CHO cells from 79.6 to 5.8 percent in response to oxidative stress^54^. Another example of transgenic protein expression for improved mammalian stress tolerance includes expression of the zebrafish zfHsp27 protein in mammalian fibroblasts for heat shock protection^55,56^. Further, there has been recent interest in improving animal cell stress tolerance through extremophile transgenes expression; for instance, the expression of brine shrimp late embryogenesis abundant proteins in fruit flies for a 20% increase in hyperosmotic stress resistance^57^. Tardigrades (*Ramazzottius varieornatus*) are microscopic animals that manifest remarkable tolerance for extreme pressure, radiation, desiccation, and temperature. While incompletely understood, there are known tardigrade-specific families of proteins that contribute to this tolerance^58^; many are thought to be intrinsically disordered, but X-ray crystallography studies have found ordered structures in at least one case^59^. Transgenic expression of tardigrade-derived proteins in human cell lines is a growing field, with several reports of improved stress tolerance^60–65^. Of particular relevance to the current work, the tardigrade mitochondrial abundant heat soluble (MAHS) protein confers osmotic stress resistance to HEp-2 cells with no significant change to gross cell morphology, but its performance remains unevaluated in cell models representative of therapy, for example ASCs^60^.

Herein, we evaluate the transgenic expression of the MAHS protein in ASCs for multi-stress tolerance. Specifically, we quantify cell survival following the representative stresses of DMSO exposure, injection stress, and metabolic stress in MAHS-expressing and control ASCs. Notably, we describe the preferential promotion of osteogenic differentiation over adipogenic differentiation in MAHS-transgenic ASCs, as measured by alkaline phosphatase activity and uptake of the neutral lipid droplet marker BODIPY following up to 14 day culture in differentiation media. However, treatment with excess glucose is sufficient to restore comparable BODIPY positive area in MAHS-expressing ASCs. This demonstration of increased stress tolerance has the potential to improve viability and efficiency of cell therapies and motivates future studies of MAHS expression in other cell types.

## Materials and methods

### Routine cell culture

All cell types were routinely cultured at 37 °C and 5% CO_2_. hTERT immortalized adipose-derived mesenchymal stem cell (ASC52telo; ATCC SCRC-4000, Manassas, VA) parental and transgenic cell lines were cultured in mesenchymal stem cell basal medium (ATCC PCS-500-030) and supplemented with mesenchymal stem cell growth kit for adipose and umbilical-derived mscs—low serum (ATCC PCS-500-040): components and final concentrations include rh FGF basic [5 ng/mL], rh FGF acidic [5 ng/mL], rh EGF [5 ng/mL], fetal bovine serum (2%) and l-Alanyl-l-Glutamine [2.4 mM]. Cells were regularly passaged at ~ 80% confluence, passage three cells were used for transduction, and all experiments were performed with cells under 20 passages from transduction.

### Cloning and lentiviral transduction

AcGFP1 control (pAcGFP1-N1 was a gift from Michael Davidson; Addgene #54705; http://n2t.net/addgene:54705; RRID:Addgene_54705) and MAHS fusion (pAcGFP1-N1-MAHS was a gift from Takekazu Kunieda; Addgene #90034; http://n2t.net/addgene:90034; RRID:Addgene_90034) genes were independently cloned into the 2nd-generation lentiviral transfer vector (pCDH-CMV-MCS-EF1α-Puro; Systems Biosciences, Palo Alto, CA). In both cases, double restriction digests were performed (EcoRI and NotI for AcGFP1-N1; NheI and NotI for AcGFP1-N1-MAHS). Restriction products were purified using horizontal agarose gel electrophoresis followed by E.Z.N.A. gel extraction kit cleanup (Omega Bio-tek; Norcross, GA). The genes of interest were separately ligated in pCDH-CMV-MCS-EF1α-Puro using T4 DNA ligase (New England Biolabs, Ipswich, MA). Ligation products were independently transformed into chemically competent DH5α (Mix & Go! competent cells; Zymo research, Irvine, CA). Transformed cells were plated on LB agar with ampicillin and cultured overnight at 37 °C. Bacterial colonies were selected for inoculation of liquid LB broth and cultured overnight at 37 °C, followed by plasmid purification (E.Z.N.A. plasmid DNA mini kit I; Omega Bio-tek). Sequences of the pCDH-CMV-AcGFP1-N1-EF1α-Puro and pCDH-CMV-AcGFP1-N1-MAHS-EF1α-Puro were confirmed using Sanger sequencing (Retrogen Inc., San Diego, CA).

HEK293TN cells were cultured to 80% confluence in DMEM with 4.5 g/L glucose, l-glutamine & sodium pyruvate, 10% fetal bovine serum, 1% penicillin/streptomycin, and 1 µg/mL amphotericin B incubated at 37 ℃ and 5% CO_2_. HEK293TN cultures were refed with fresh media and then triple transfected with the transfection reagent Endofectin Max (GeneCopoeia; Cat# EF013), packaging plasmid psPAX2 (Addgene #12260), envelope plasmid pMD2.G (Addgene #12259), and either lentiviral transfer vector pCDH-CMV-AcGFP1-N1-EF1α-Puro or pCDH-CMV-AcGFP1-N1-MAHS-EF1α-Puro and then incubated for 48 h at 37 ℃ and 5% CO_2_. Viral supernatant was collected at 48 h and filtered prior to addition to media of routinely cultured ASCs (ASC52telo; ATCC). Cells were selected using 1 µg/mL puromycin until a pure population was observed via fluorescence microscopy.

### Mitochondrial localization and morphology analysis

AcGFP1- and MAHS-transgenic ASCs were seeded at an initial density of 10,000 cells/well in routine culture media. After 24 h mitochondrial localization and morphology was assessed. Four sample replicates of each genotype were obtained. Samples were stained with 125 nM MitoView Fix 640 (Biotium #70082, Fremont, CA) in cell culture media. After 2 h, samples were fixed for 5 min with 4% paraformaldehyde (ThermoFisher #T353500, Riverside, CA) in PBS and subsequently permeabilized for 5 min with 0.25% Triton X-100 (ThermoFisher #BP151500, Riverside, CA) in PBS. Samples were incubated overnight at 4 °C in a stain solution of [1:750] DyLight Phalloidin 554 (Cell Signaling Technology #13054, Danvers, MA) in 1× DAPI (Invitrogen #D1306, Waltham, MA). Samples were washed three times with PBS and stored in PBS at 4 °C before imaging. All samples were imaged using a DFC9000 GT sCMOS camera on a DMi8 inverted microscope (Leica Microsystems, Chicago, IL). A 64-position tile scan of ~ 0.95 cm^2^ area was obtained for each sample with 1 µm steps to focus each image. Four separate cell platings were used as biological replicates.

A custom MATLAB script (MATLAB R2022a; MathWorks, Natick, MA) was written to quantify the Pearson correlation coefficient between expressed protein localization (AcGFP1 or MAHS) and mitochondrial morphology (MitoView). For all analysis, quantification of each experimental group was obtained from averaged cell counts of 64 positions per sample. Mitochondrial morphology characteristics were quantified using the MiNA software^66^. Pairwise comparison between Pearson correlation coefficients and mitochondrial morphology characteristics of AcGFP1- and MAHS-expressing ASCs were performed using two-sample *t*-tests with significance of α = 0.05 (MATLAB). Medians and individual data points are indicated by black bars and triangles, respectively. The dataset utilized for generating all graphical representations and conducting statistical analyses is available in the supplementary materials.

### Media depletion tolerance

Experiments were performed in 48-well plates. AcGFP1- and MAHS-expressing ASCs were seeded at an initial density of 10,000 cells/well in routine culture media. After 24 h, culture media was replaced with media diluted with various concentrations of DPBS—no further media changes were performed following initial administration. DPBS concentrations evaluated in ASC52telos over a period of 72 h include 0%, 70%, 80%, and 90% DPBS. Cells were fixed as described above, incubated in DAPI and [1:750] DyLight Phalloidin 554 solution and imaged as described above. Three separate cell platings were used as biological replicates. Viability analysis was also performed using viability/cytotoxicity assay kit (#30002; Biotium, Fremont, CA) solutions diluted to final concentrations of Calcein-AM [2 µM] and EthD-III [4 µM] in PBS. However, dead cell loss due to cell detachment over the extended culture time resulted in high live/dead ratios not representative of the cell loss (Figure S1B). Consequently, the cell population remaining attached was considered as the primary metric of media depletion tolerance.

A custom MATLAB script was written to quantify DAPI counts within phalloidin cell borders and express outputs as a percentage of cell viability normalized to respective control AcGFP1 or MAHS samples. For all analysis, quantification of each experimental group was obtained from averaged cell counts of 64 positions per sample. Analysis of the extent to which the relationship between DPBS treatment and cell population varied with genotype was performed with N-way analysis of variance (ANOVAN). Post hoc comparisons at each tested DPBS concentration were performed using Tukey’s honestly significant difference (Tukey’s HSD) test. All statistical tests were performed in MATLAB. Statistical tests and data visualization were performed in MATLAB as described above. The dataset utilized for generating all graphical representations and conducting statistical analyses is available in the supplementary materials.

### Freezing tolerance

AcGFP1- and MAHS-expressing ASCs were routinely passaged, resuspended in 100% bovine calf serum (BCS, Fisher Scientific #SH3007303HI, Hampton, NH) and deposited directly into liquid nitrogen (flash freezing) or in a cell freezing container for controlled freezing (− 1 °C/min) down to − 80 °C. After 7 days, cryovials were removed from liquid nitrogen storage, centrifuged, and resuspended in viability/cytotoxicity assay kit (#30002; Biotium, Fremont, CA) solutions diluted to final concentrations of Calcein-AM [2 µM] and EthD-III [4 µM] in PBS. Samples were transferred into 24 well plates and incubated for 30 min at room temperature. Cells were imaged as described above. Three separate cell suspensions were used as biological replicates.

A custom MATLAB script was written to quantify cell viability. For all analysis, quantification of each experimental group was obtained from averaged cell counts of 36 positions per sample. Pairwise comparisons at each tested cryopreservation period were performed using two sample *t*-tests. All statistical tests were performed in MATLAB. Statistical tests and data visualization were performed in MATLAB as described above. The dataset utilized for generating all graphical representations and conducting statistical analyses is available in the supplementary materials.

### DMSO tolerance

Experiments were performed in 48-well plates. AcGFP1- and MAHS-expressing ASCs were seeded at an initial density of 10,000 cells/well in routine culture media. After 24 h the cells were treated with varying concentrations of dimethyl sulfoxide (DMSO; Gaylord Chemical Company #MFCD00002089, Covington, LA). Cells were treated for 72 h with 0%, 1%, 1.5%, 2%, 2.5%, 3%, 3.5%, 4%, and 5% DMSO. Cells were incubated in DAPI and [1:750] DyLight Phalloidin 554 solution and imaged as described above. Three separate cell platings were used as biological replicates. Viability analysis was also performed using viability/cytotoxicity assay kit (#30002; Biotium, Fremont, CA) solutions diluted to final concentrations of Calcein-AM [2 µM] and EthD-III [4 µM] in PBS. Similar to media depletion tolerance testing, detachment of dead cells led to live/dead ratios not representative of cell loss (Figure S2B). Consequently, cell population was considered as the primary metric of DMSO tolerance.

The custom MATLAB script was used to quantify DAPI counts and calculate relative cell population as described above. Analysis of the extent to which the relationship between DMSO treatment and cell viability varied with genotype was performed with analysis of covariance (ANCOVA) and Tukey’s honestly significant difference (Tukey’s HSD) test were conducted as described above. The dataset utilized for generating all graphical representations and conducting statistical analyses is available in the supplementary materials.

### Biomaterial fabrication

9 g of Sylgard 184 silicone elastomer base and 1 g of silicone curing agent (WPI SYLG184, Sarasota, FL) were vigorously mixed and placed in a desiccator vacuum chamber for 30 min to remove air bubbles. The solution was then poured into a 90 mm petri dish and cured in an oven at 60 °C for 2 days to prevent upstream solvent leaching into cell culture. ¼” diameter circular holes were cut into cured polydimethylsiloxane (PDMS), and PDMS “barriers” were firmly pressed onto 22 × 22 mm glass coverslips. PDMS barriers and coverslips were sterilized by autoclave and utilized for injection tolerance experiments.

### Injection tolerance

Prior to injection, AcGFP1- and MAHS-transgenic ASCs were washed three times with DPBS (+ , +), routinely passaged and resuspended at a density of 5000 cells/µL in viability/cytotoxicity assay kit (Biotium #30002, Fremont, CA) solutions diluted to final concentrations of Calcein-AM [2 µM] and EthD-III [4 µM] in PBS. The resuspended solutions were immediately injected onto glass coverslips surrounded by PDMS barriers at an injection rate of 1,000 µL/min. Samples were imaged on an inverted microscope as described above. A 36-position tile scan of ~ 0.53 cm^2^ area was obtained for each sample with 1 µm steps to focus each image. Six cell platings were used as biological replicates for each experimental group.

The custom MATLAB script was used to calculate percentage of Calcein-AM-expressing cells as described above. Analysis of the extent to which the relationship between needle gauge and cell viability varied with genotype was performed through 2-way analysis of variance (2-way ANOVA) and Tukey’s honestly significant difference (Tukey’s HSD) test as described above. The dataset utilized for generating all graphical representations and conducting statistical analyses is available in the supplementary materials.

### Adipogenic differentiation

Experiments were performed in 24-well plates. AcGFP1- and MAHS-expressing ASCs were seeded at an initial density of 10,000 cells/well in routine culture media. After 24 h, cells were treated with adipogenic media for 7, 14, and 21 days with media changes every 3 days. Adipogenic media components include DMEM/F-12 50-50 base media (Corning #15-090-CM, Corning, NY), fetal bovine serum (3%, Gibco #A5256701, Billings, MT), biotin [200.56 mM] (Sigma–Aldrich #B4501, St. Louis, MO), IBMX [100 mM], (Tocris Bioscience #284550, Bristol, UK), indomethacin [20 mg/mL] (Santa Cruz Biotechnology #SC-200503, Dallas, TX), insulin [5 mg/mL] (PeproTech #100-11, Cranbury, NJ), dexamethasone [1 mM], (Sigma–Aldrich #50-02-2, St. Louis, MO), and d-calcium pantothenate [671.53 mM] (ThermoFisher Scientific #AC416750000, Riverside, CA). As separate experiments, cells were treated with regular or adipogenic media with 0 mM, 5 mM, 10 mM, 20 mM, 30 mM, and 40 mM glucose (d-( +)-Glucose; Fisher Scientific #MFCD00148912, Hampton, NH). Samples were fixed for 5 min with 4% paraformaldehyde and incubated for 30 min at room temperature in the dark in [4.5 µM] BODIPY 493/503 (molecular probes #D3922, Eugene, OR) in blocking buffer. Cells were imaged as described above. Three separate cell platings were used as biological replicates.

A custom MATLAB script was written to quantify adipogenic expression in terms of percent of cellular BODIPY positive area. For all analysis, quantification of each experimental group was obtained from averaged cell counts of 64 positions per sample. Analysis of the extent to which the relationship between culture period and BODIPY positive area varied with genotype was performed through was performed through N-way analysis of variance (ANOVAN) and Tukey’s honestly significant difference (Tukey’s HSD) test as described above. The dataset utilized for generating all graphical representations and conducting statistical analyses is available in the supplementary materials.

### Osteogenic differentiation

Experiments were performed in 24-well plates. AcGFP1- and MAHS-expressing ASCs were seeded at an initial density of 10,000 cells/well in routine culture media. After 48 h cells were treated with osteogenic media (osteocyte differentiation tool; ATCC PCS-500-052, Manassas, VA). Media was exchanged every 4 days until the culture endpoint. Samples were fixed for 5 min with 4% paraformaldehyde and incubated in the dark for 30 min at room temperature in a stain solution of naphthol AS-MX phosphate (0.1%, research organics #1596-56-1, Cleveland, OH) and fast red violet LB salt (0.1%, electron microscopy sciences #32348-81-5, Hatfield, PA) in 56 mM AMPD buffer, pH 9.9 (2-amino-2-methyl-1,3-propanediol, TCI America #A0332, Portland, OR). Samples were washed three times with PBS and stored in PBS at 4 °C before imaging. Representative samples were captured with a brightfield microscope with MU300 digital camera (AmScope, Irvine, CA). Three separate cell platings were used as biological replicates.

A custom MATLAB script was adapted from a published color deconvolution script to quantify osteogenic expression in terms of the mean pixel intensity (MPI) of cellular alkaline phosphatase activity^67,68^. For all analysis, quantification of each experimental group was obtained from averaged MPIs of six representative brightfield images per sample. Pairwise comparisons at each tested culture period were performed using two-sample *t*-tests. All statistical tests were performed in MATLAB as described above. The dataset utilized for generating all graphical representations and conducting statistical analyses is available in the supplementary materials.

## Results

### MAHS expression in ASCs is localized to the mitochondria

**Figure 1:**
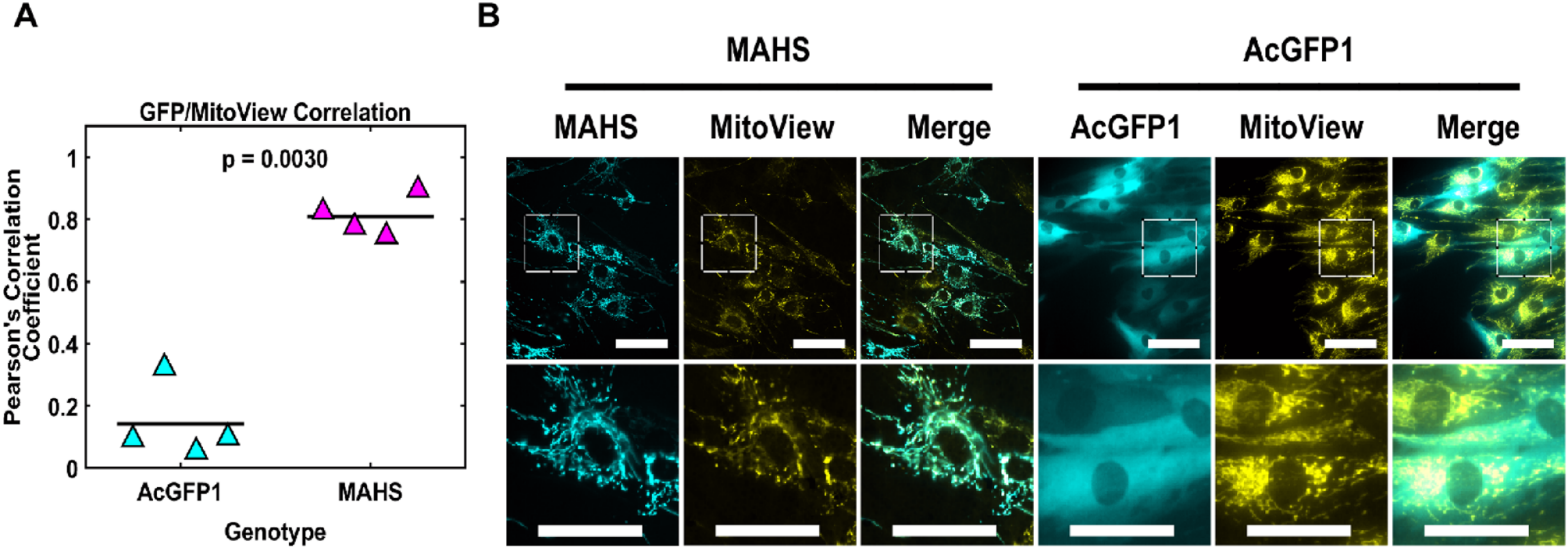
Mitochondrial localization of MAHS. (**A**) Quantification of Pearson correlation coefficient for the overlap between expressed protein localization and mitochondrial morphology (two-sample *t*-test*, n* = 4). The denoted p-value indicates the pairwise difference in GFP/MitoView correlation between the genotypes. (**B**) Maximum projections of the overlap between expressed protein localization (AcGFP1 or AcGFP1-MAHS, cyan) and mitochondrial morphology (MitoView, yellow) in ASC52telos transduced with either the AcGFP1 or AcGFP1-MAHS transgene. Scale bars are 25 µm.

We first assessed the localization of MAHS expressed in ASCs. Fluorescent localization allows for confirmation of the expression of MAHS in ASCs (Fig. 1). Qualitatively, AcGFP1-tagged MAHS was observed to localize to the mitochondria in ASCs, while AcGFP1 alone (control) was expressed nonspecifically throughout the cytosol. Quantitatively, MAHS expression and MitoView had a significantly larger Pearson correlation coefficient than AcGFP1 and MitoView. These findings are consistent with transient expression studies of MAHS in human HEp-2 and HEK293T cells^60^. We did not detect any substantial difference in mitochondrial morphology between cell genotypes, using the mitochondrial network analysis (MiNA) software to assess several morphometric parameters (Figure S3A-K)^66^. We also did not detect any change in MitoView intensity in MAHS-transgenic ASCs(Figure S3L).

### MAHS confers DMSO tolerance to ASCs

**Figure 2:**
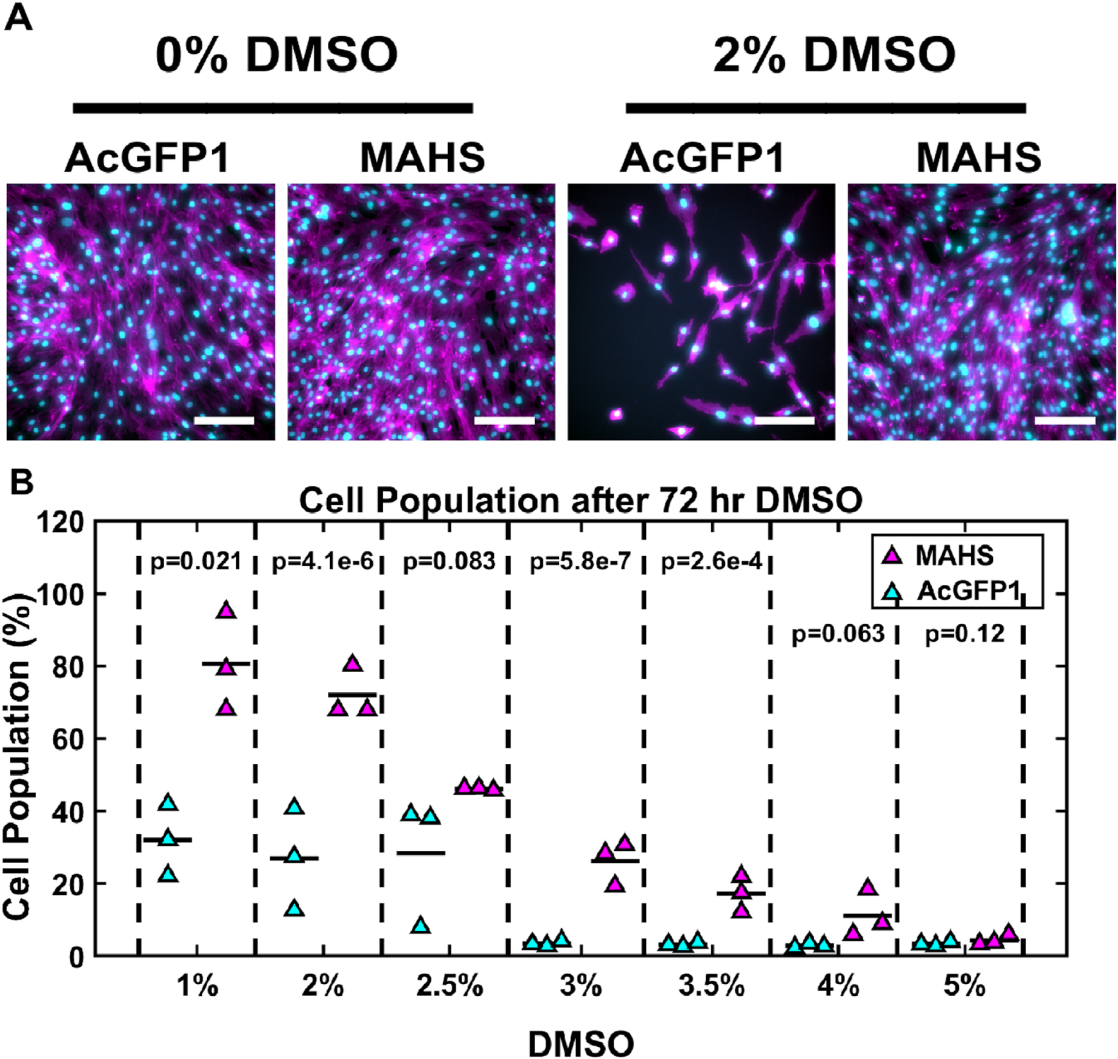
DMSO stress. (**A**) Maximum projections of the nuclear (DAPI, cyan) and cytoskeletal (phalloidin, magenta) structures of ASC52telos transduced with either the AcGFP1 or MAHS transgene following 72 h of DMSO treatment. Scale bars are 200 µm. (**B**) Quantification of cell population in AcGFP1- and MAHS-expressing ASCs following a 72 h DMSO treatment (ANCOVA, Tukey’s HSD, *n* = 3). Cell population quantification was normalized to cells cultured without DMSO for 72 h. Denoted p-values indicate pairwise difference in cell population between the genotypes at each specific DMSO concentration.

To assess the effect of MAHS expression on DMSO tolerance, ASCs were incubated in culture media containing various concentrations of DMSO for 72 h. Cell population after DMSO treatment, as measured by cell count normalized to control (0%) treatment across 1–5% treatment doses, was significantly higher in MAHS-transgenic ASCs than in AcGFP1-transgenic ASCs (ANCOVA, p = 0.0033). Further, survival was increased in MAHS-expressing ASCs in media with 1% (49% ± 11%), 2% (45% ± 8.8%), 3% (23% ± 5.4%), and 3.5% (14% ± 5.2%) DMSO (Fig. 2). These data suggest a MAHS-influenced tolerance to chronic exposure to low amounts of DMSO. We also tested live-dead viability under these treatment conditions (Figure S2); however, cell population was a more appropriate stress metric due to artificial inflation of live cell percentages resulting from cell loss over the 72 h treatment period. Relatedly, we tested viability after cryopreservation without DMSO using both slow and flash freezing. However, MAHS did not offer improved survival in this context (Figure S4**)**.

### MAHS confers injection tolerance to ASCs

**Figure 3:**
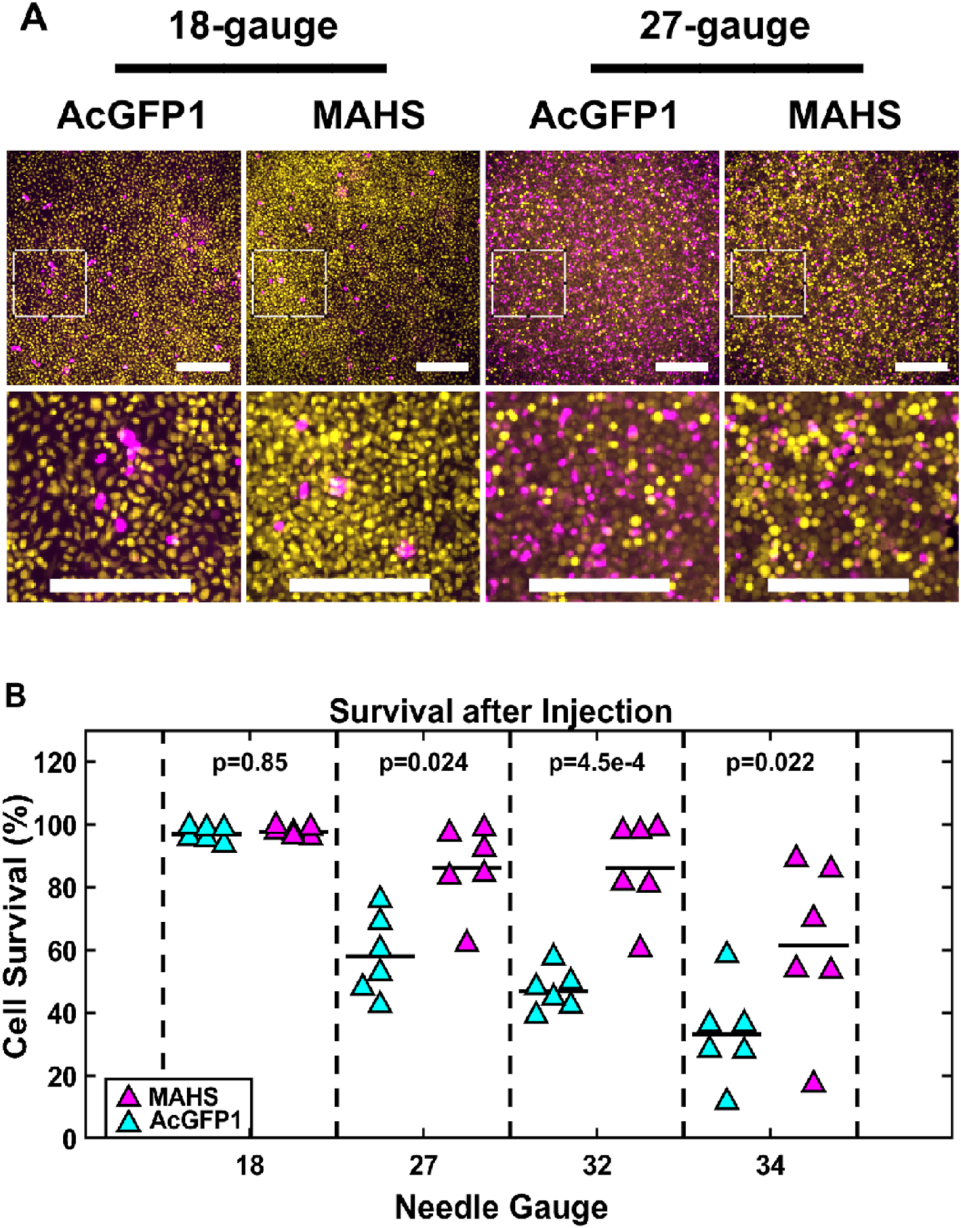
Injection shear stress. (**A**) Maximum projections of live (Calcein-AM, yellow) and dead (EthD-III, magenta) AcGFP1- and MAHS-expressing ASC52telos following injection through various needle gauges. Scale bars are 300 µm. (**B**) Quantification of cell survival in AcGFP1- (cyan) and MAHS-transgenic (magenta) ASCs following injection through various needle gauges (2-way ANOVA, Tukey’s HSD, *n* = 6). Denoted p-values indicate pairwise difference in cell survival between the genotypes at each specific needle gauge.

We quantified cellular survival in response to injection-induced shear stress, as measured by live/dead cell ratio following 5000 cell/µL injections through 18-34 gauge needles at 1 mL/min, which are relevant for clinical injections^27,32,33,69^. Cell survival, as measured by percentage of live cells, was increased in MAHS-expressing ASCs following injection through 27-gauge (28% ± 13%), 32-gauge (39% ± 11%), and 34-gauge (28% ± 21%) needles (Fig. 3). These data indicate MAHS expression increases tolerance for injection stress in ASCs.

### MAHS confers metabolic stress tolerance to ASCs

**Figure 4:**
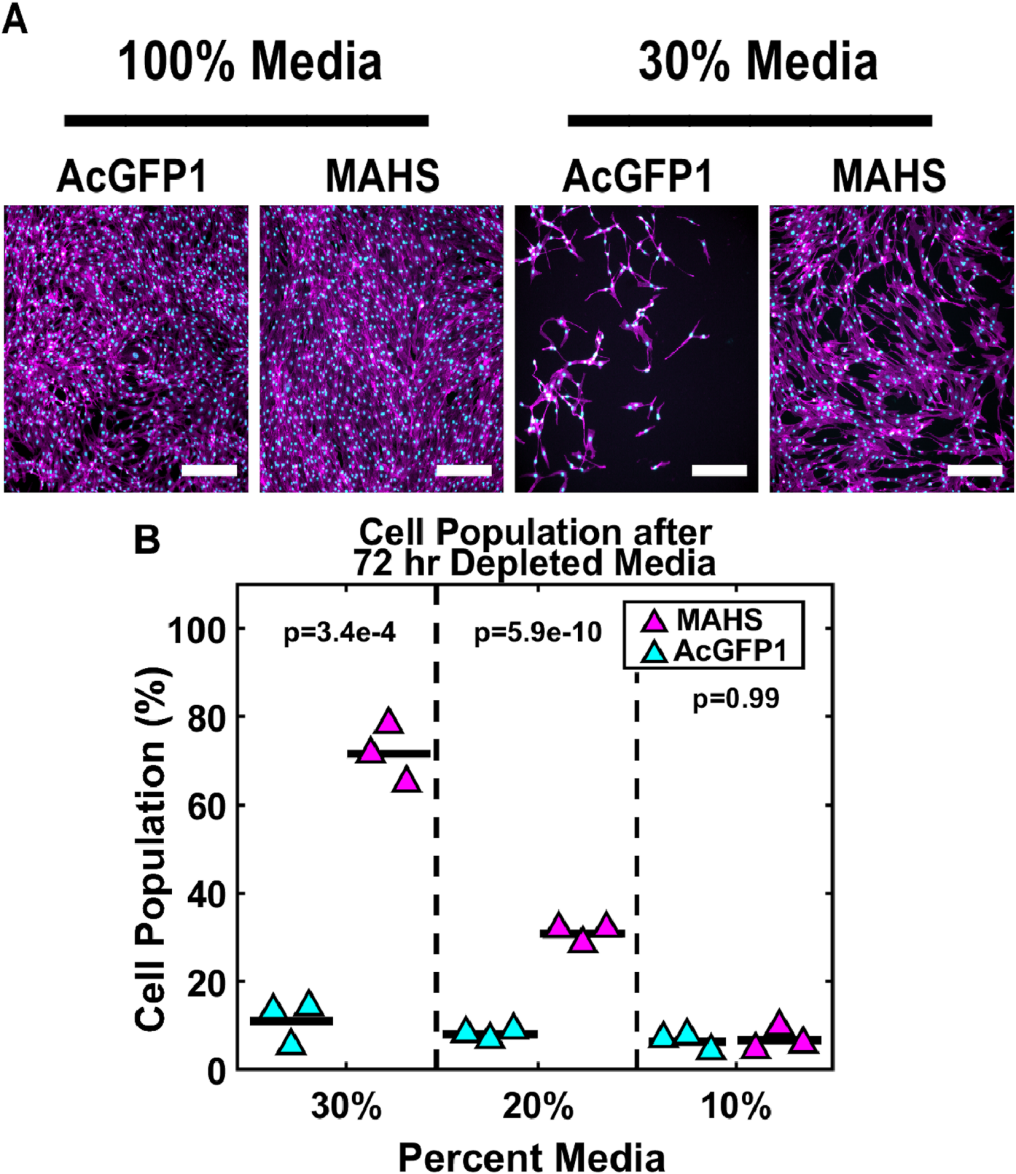
Metabolic stress. (**A**) Maximum projections of the nuclear (DAPI, cyan) and cytoskeletal (phalloidin, magenta) structures of ASC52telos transduced with either the AcGFP1 or MAHS transgene following 72 h of media depletion. Scale bars are 300 µm. (**B**) Quantification of cell population in AcGFP1- and MAHS-expressing ASCs following a 72 h media depletion (ANOVAN, Tukey’s HSD, *n* = 3). Cell survival quantification was normalized to cells cultured in 100% media for 72 h. Denoted p-values indicate pairwise difference in cell population between the genotypes at each specific media concentration.

To analyze the effect of MAHS expression on ASC population in response to prolonged nutrient depletion, ASCs were incubated in culture media diluted to 30%, 20%, or 10% with DPBS containing calcium and magnesium for 72 h and assessed via cell counts compared to full media controls (Fig. 4). There was a significant protective effect of MAHS expression against media depletion groups (ANOVAN, p = 2.0e-9). Further, cell population was significantly higher in MAHS-transgenic ASCs than in AcGFP1-transgenic ASCs in 30% (61% ± 8.6%) and 20% (23% ± 1.0%) media (α < 0.05, Tukey’s HSD). These data suggest that MAHS expression aids in metabolic stress resistance in ASCs. We also tested live-dead viability under these treatment conditions (Figure S1); however, as previously mentioned, substantial cell detachment over the 72 h treatment period skewed live cell measurements, and cell population was instead considered as the primary stress metric.

### MAHS-transgenic ASCs preferentially differentiate down osteogenic over adipogenic lineages

**Figure 5:**
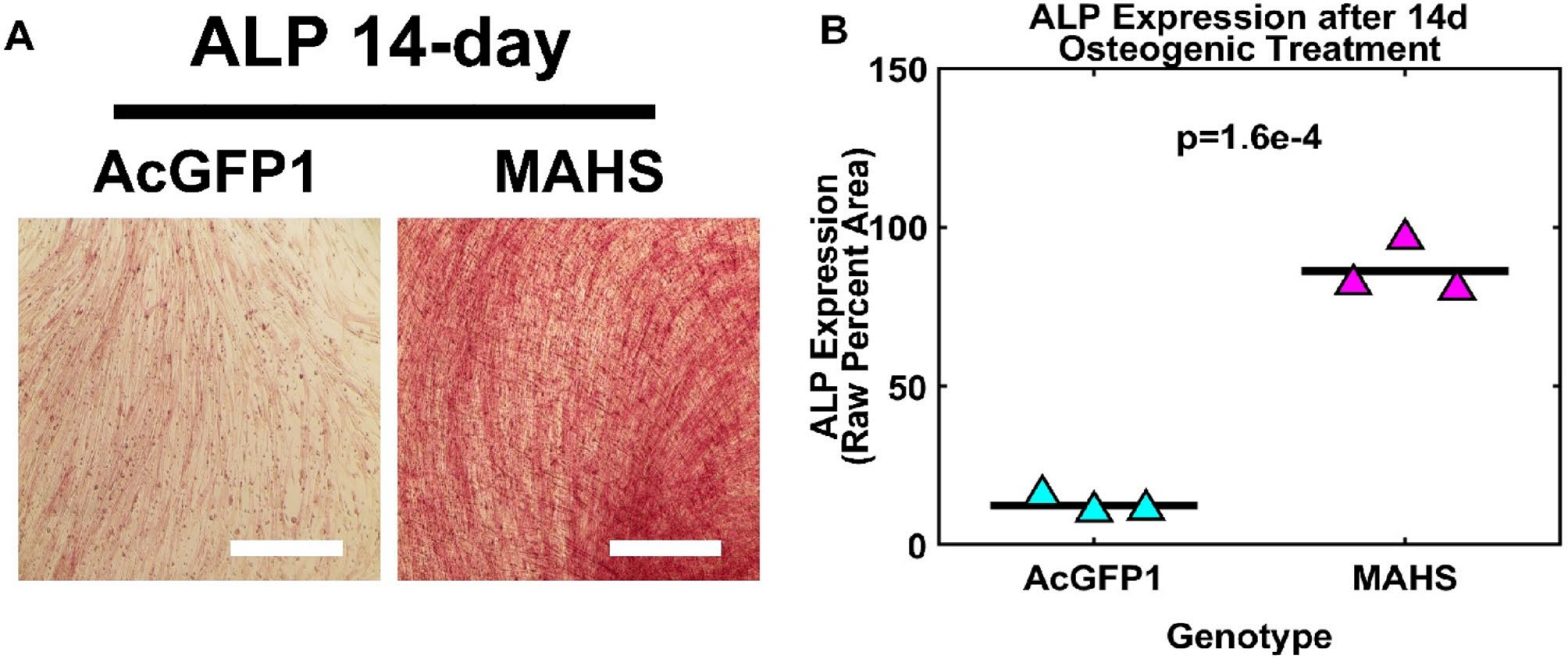
Osteogenic differentiation. (**A**) Brightfield images of 14 day alkaline phosphatase expression (ALP, scale bars = 300 µm) in AcGFP1- and MAHS-transgenic ASCs. (**B**) Quantification of 14 day ALP expression (two-sample *t*-test, *n* = 3). The denoted p-value indicates the pairwise difference in ALP expression between the genotypes.

**Figure 6:**
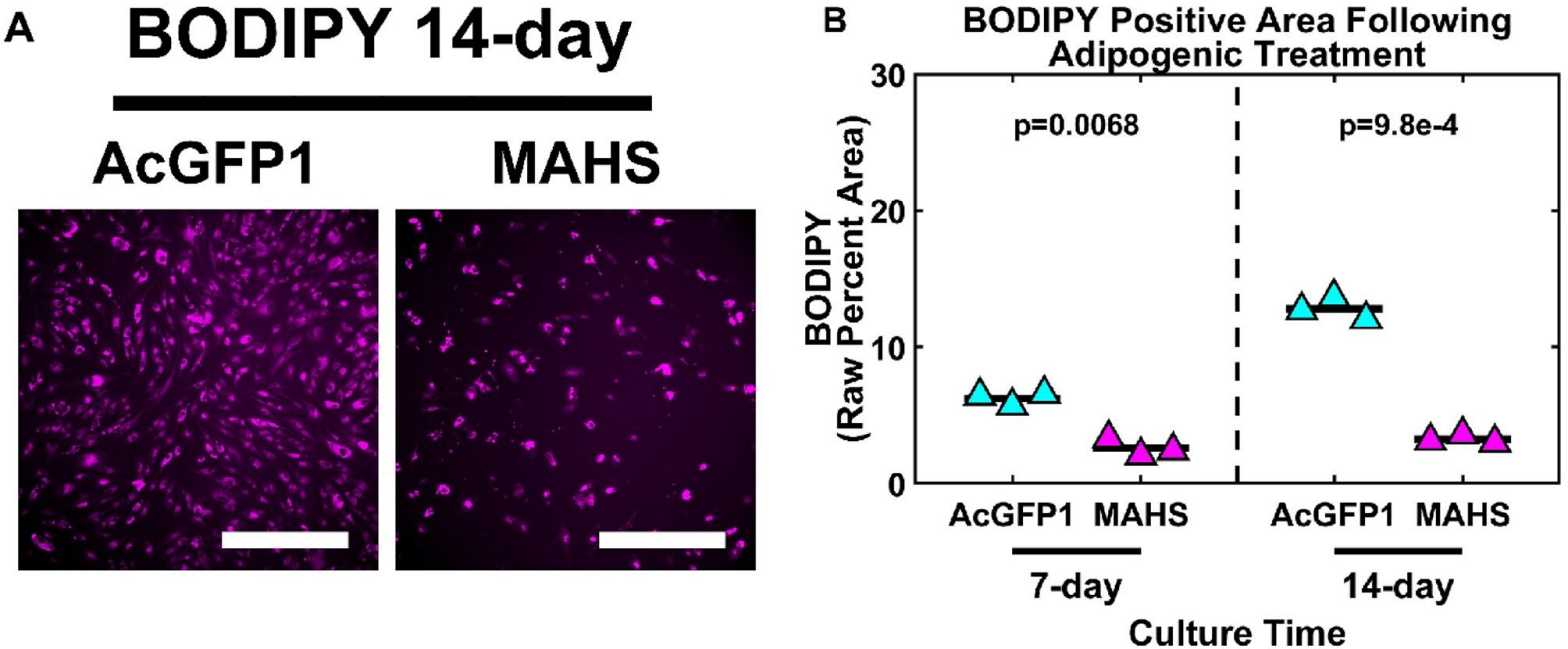
Adipogenic differentiation. (**A**) Maximum projections of 14 day BODIPY positive area (BODIPY, scale bars = 250 µm) in AcGFP1- and MAHS-transgenic ASCs. (**B**) Quantification of 14 day BODIPY positive area (two-sample *t*-test, *n* = 3). Denoted p-values indicate pairwise difference in BODIPY positive area between the genotypes at each specific time point.

Stem cell therapies may rely on differentiation into specific cell types; this may be influenced by the expression of an exogenous protein. To test the effect of MAHS expression on ASC differentiation, we used standard methods to induce either adipogenic or osteogenic differentiation^70^. Osteogenic differentiation, as measured by quantification of the early osteogenic marker alkaline phosphatase (ALP) activity, was significantly higher in MAHS-expressing ASCs than in AcGFP1-expressing ASCs following a 14 day culture period (73.7510% ± 6.5310%) in osteogenic media (Fig. 5). Conversely, adipogenic differentiation, as measured by quantification of the neutral lipid droplet marker BODIPY, was significantly higher in AcGFP1-expressing ASCs than in MAHS-expressing ASCs following 7 day (3.644% ± 0.5228%) and 14 day (9.568% ± 0.5199%) culture periods in adipogenic media (Fig. 6). These data suggest that MAHS expression affects ASC differentiation in favor of an osteogenic lineage over an adipogenic lineage.

### Excess glucose partially rescues adipogenic differentiation capacity in MAHS-transgenic ASCs

**Figure 7:**
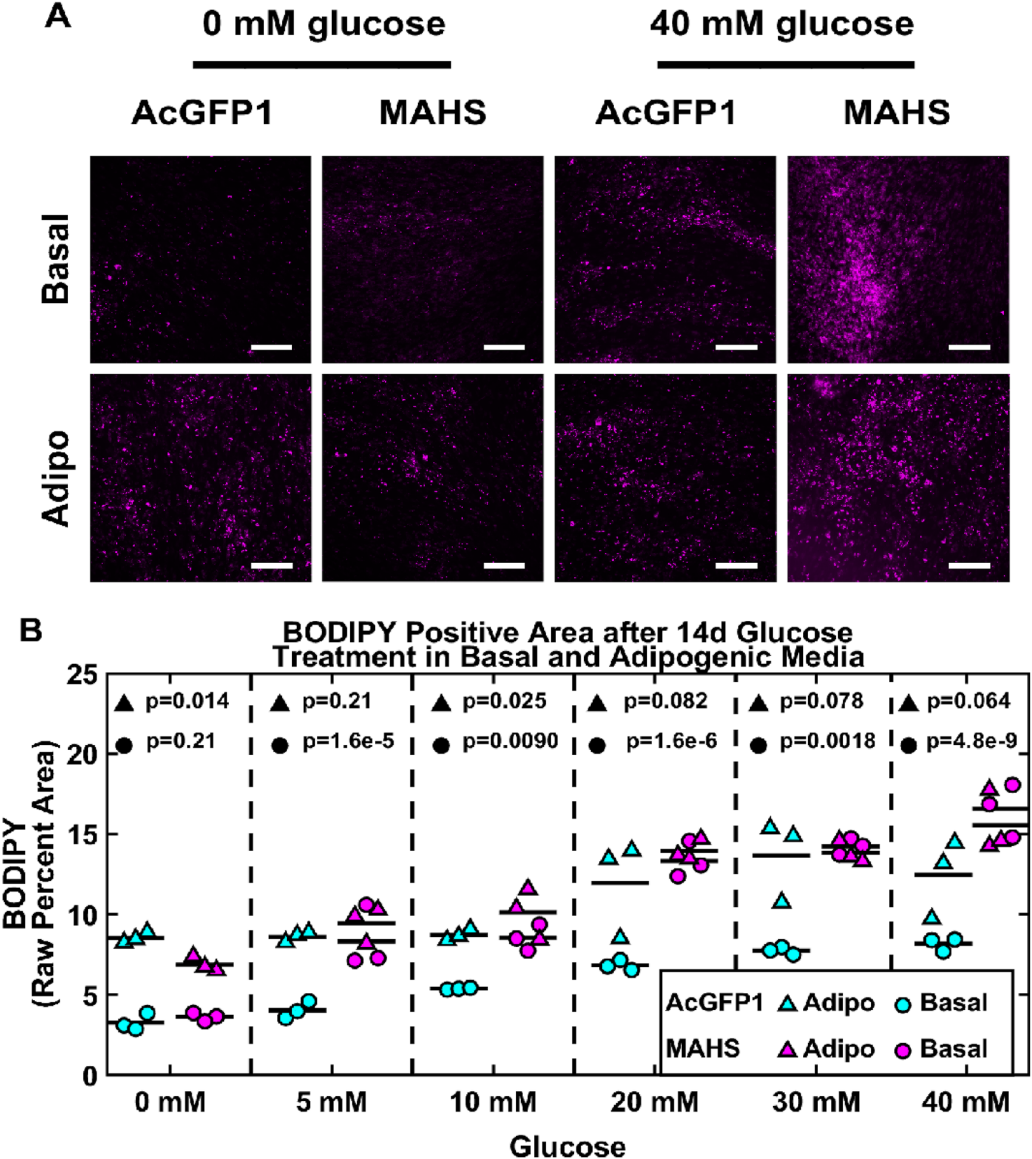
Adipogenic differentiation (14 day glucose-treated basal and adipogenic media). (**A**) Maximum projections of 14 day BODIPY positive area (BODIPY, scale bars = 250 µm) in AcGFP1- and MAHS-transgenic ASCs following culture in glucose-treated basal and adipogenic media. (**B**) Quantification of 14 day BODIPY positive area in glucose-treated basal and adipogenic media in AcGFP1- and MAHS-expressing ASCs (ANOVAN, Tukey’s HSD, *n* = 3). Denoted p-values indicate pairwise difference in BODIPY positive area between the genotypes at each specific glucose concentration.

**Figure 8:**
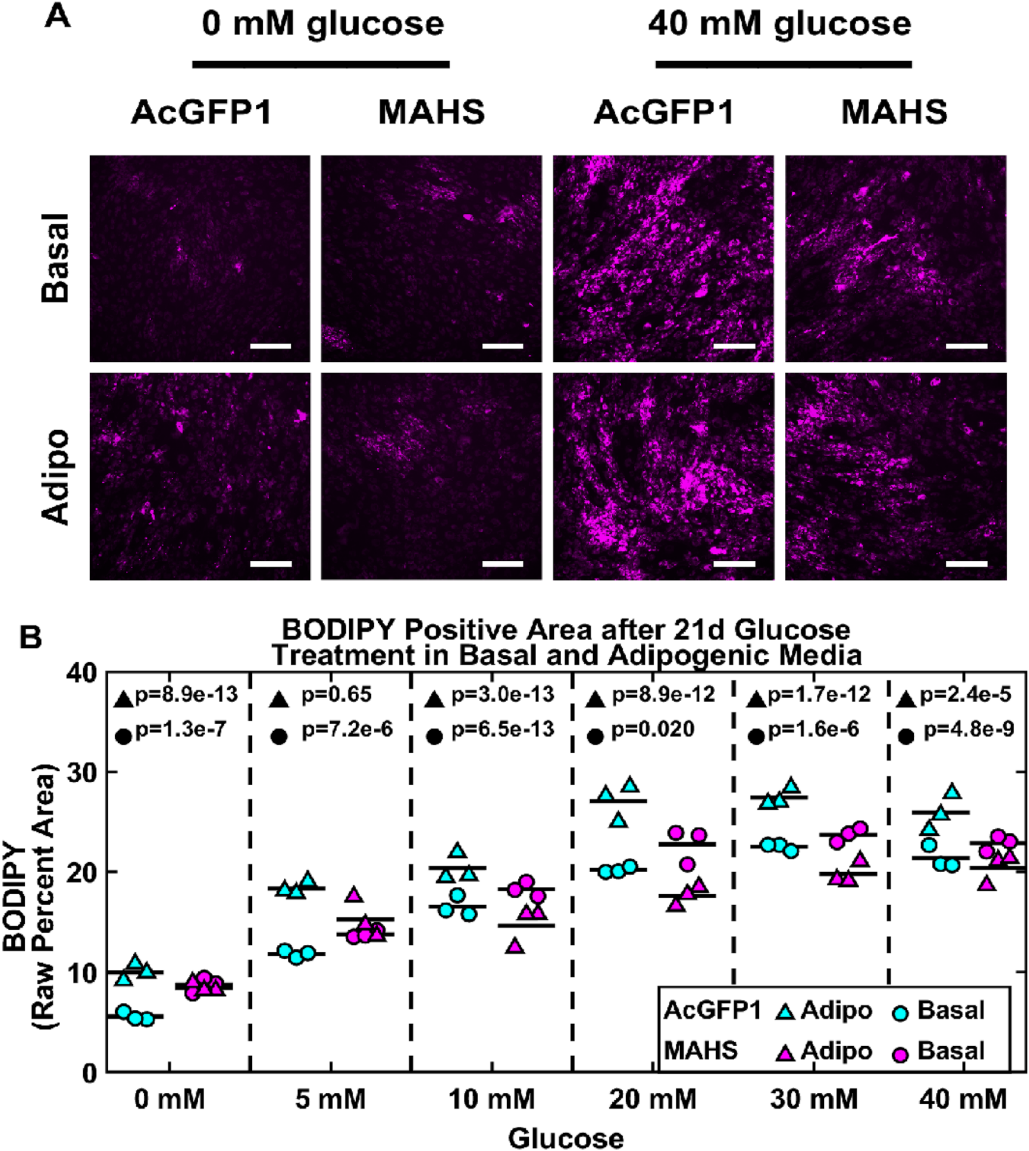
Adipogenic differentiation (21 day glucose-treated basal and adipogenic media). (**A**) Maximum projections of 21 day BODIPY positive area (BODIPY, scale bars = 250 µm) in AcGFP1- and MAHS-expressing ASCs following culture in glucose-treated basal and adipogenic media. (**B**) Quantification of 21 day BODIPY positive area in glucose-treated basal and adipogenic media in AcGFP1- and MAHS-transgenic ASCs (ANOVAN, Tukey’s HSD, *n* = 3). Denoted p-values indicate pairwise difference in BODIPY positive area between the genotypes at each specific glucose concentration.

To determine whether adipogenic differentiation capacity could be rescued in MAHS-expressing ASCs, we cultured AcGFP1- and MAHS-transgenic ASCs in basal and adipogenic media supplemented with up to 40 mM glucose, a known AMPK inhibitor^71^, over 14 day (Fig. 7) and 21 day (Fig. 8) culture time points. Interestingly, we noted an increase in BODIPY positive area in both 14 day (5 mM: 4.289% ± 1.432%; 10 mM: 3.159% ± 0.723%; 20 mM: 6.507% ± 0.8103%; 30 mM: 6.500% ± 0.275%; 40 mM: 8.354%) and 21 day (0 mM: 3.158% ± 0.3629%; 5 mM: 1.965% ± 0.000800%; 10 mM: 1.731% ± 0.277%; 20 mM: 2.567% ± 1.477%; 30 mM: 1.216% ± 0.328%; 40 mM: 1.492% ± 0.3698%) basal media cultures of MAHS-expressing ASCs supplemented with up to 40 mM glucose relative to control ASCs. Conversely, MAHS-expressing ASC BODIPY positive area was reduced relative to control ASCs under 21 day adipogenic media culture conditions supplemented with up to 40 mM glucose (0 mM: 1.530% ± 0.3840%; 5 mM: 3.07% ± 1.386%; 10 mM: 5.6967% ± 0.5236%; 20 mM: 9.406% ± 0.8621%; 30 mM: 7.602% ± 0.2238%; 40 mM: 5.529% ± 0.3288%), but with significant increases in BODIPY positive area compared to 21 day MAHS-expressing ASC adipogenic media culture conditions with no extra glucose added. BODIPY positive area was largely constant across cell genotypes cultured in adipogenic media supplemented with glucose over 14 days.

## Discussion

### Transgene localization to mitochondria

In tardigrades, the MAHS protein plays a critical role in guarding against desiccation-related mitochondrial damage, and its protective activity against metabolic stress associated with hyperosmotic conditions is documented in HEp-2 cells^60^. However, potential for application of MAHS expression in mammalian cells for therapeutic purposes is currently limited due to the lack of knowledge of whether MAHS performs similar protective functions in therapeutic cells such as ASCs. Here, we demonstrated that the AcGFP1-tagged MAHS protein is stably expressed in ASCs and is localized to the mitochondria (Fig. 1). This is consistent with other studies of in vitro expression of MAHS in mammalian cells^60^. Although direct experimental data is currently lacking, it has been hypothesized that MAHS may stabilize the two mitochondrial phospholipid bilayers by maintaining appropriate spacing between polar head groups of membrane phospholipids^58^, this may be part of the protective mechanism in ASCs. As an intrinsically disordered protein, MAHS may undergo multiple conformational changes to secondary structure to confer multistress tolerance, similar to the *Pv*LEA-22 peptide, which transitions from an alpha-helical structure to β-sheet conformation upon contact with cell membrane structures^58,72–75^. It is important to note that MitoView 640 staining intensity is independent of mitochondrial membrane potential, unlike some varieties of mitochondrial dyes^76,77^. Therefore, while we did not observe morphological changes to mitochondria, it is possible that mitochondrial membrane potential may be altered in MAHS-expressing ASCs. If so, alteration of membrane potential may contribute to the restrained adipogenic differentiation capacity observed in MAHS-expressing ASCs, especially given the partial rescue of adipogenic differentiation in MAHS-expressing ASCs following chronic glucose treatment.

### DMSO tolerance

DMSO is a valuable mammalian cell cryoprotectant: it is commonly used to protect ASCs and other therapeutic cell types from cell membrane damage caused by intracellular ice formation during flash freezing, as well as dehydration and hyperosmotic stress due to solute concentration and solvent freezing during slow cooling^18,19^. However, prolonged exposure to DMSO compromises mitochondrial membrane integrity through generation of damaging ROS species and lowered mitochondrial membrane potential, and facilitates release of calcium from the endoplasmic reticulum to initiate apoptosis^22–26^. In addition to limiting viability of stem cells for therapeutic purposes, patients receiving injections or grafts containing these cells have been documented to experience severe DMSO-specific neurological and immunological responses^27–30^. Chronic DMSO exposure studies show that the MAHS protein provides protection to ASCs against cell damage induced by up to 3.5% DMSO (Fig. 2). Given the mitochondrial localization of MAHS and hypothesized membrane interaction^58^, it is possible that MAHS supports mitochondrial membrane integrity and reduces DMSO-induced membrane permeabilization. However, MAHS expression did not protect ASCs from stressors associated with freezing without the use of a cryoprotectant; this is contrary to the tolerance exhibited by tardigrades during freezing (Figure S4)^60,78^.

### Injection tolerance

Injection of ASCs is a treatment method for diseased, damaged, and nutrient deprived tissues. Clinical needle diameters are often reduced for patient comfort and to permit safe injection into sensitive tissues^31,36–38^. However, cell injections expose cells to high shear and extrusion stresses, resulting in viability loss. It has been reported for cell viability loss rates to be as high as 68–99% following therapeutic injection into target tissues^39–41^. Injection studies across a range of clinical needle gauges (i.e., 18-34 gauge) indicate that MAHS expression provides protection to ASCs against shear stress (Fig. 3). While the specific mechanism is out of the scope of this work, there are several potential mechanisms for this effect. In other cell types, it has been shown that fluid shear stress can directly rupture cell membranes, stimulate the release of intracellular calcium from the endoplasmic reticulum and cytochrome c from the mitochondria to initiate apoptotic signaling, and promote expression of additional apoptosis regulating genes^60,79–81^. Further, it has also been shown that damaging ROS species produced in mitochondria are implicated as secondary messengers in the modulation of shear-induced gene expression in endothelial cells^82^. As previously speculated, MAHS may be directly preventing mitochondrial membrane disruption^58^. MAHS may also indirectly strengthen the cell membrane or cortical cytoskeleton. For example, prior work has shown increased stiffness in multiple stem cell types prior to and after osteogenic differentiation^83,84^; given the propensity for osteogenic differentiation in MAHS-expressing ASCs, it is possible that these cells innately possess stiffened membranes and matrices that are resistant to deformation.

### Metabolic stress tolerance

Stem cell therapies may be injected into ischemic tissues for repair and regeneration of damaged tissue, where metabolic stress is common in response to limited oxygen and nutrient supply caused by impaired perfusion^85,86^. Specifically, starvation is known to induce oxidative stress through ROS production, decreased mitochondrial membrane potential, and activation of the apoptosis cascade through calcium release from the ER^20–24^. Here, the MAHS protein provides protection to ASCs against metabolic stress induced by nutrient depletion, suggesting potential for increased viability in damaged or ischemic engraftment sites (Fig. 4). While the specific mechanism is outside the scope of this study, potential mechanisms may include reduced energy generation or ROS tolerance. While not quantified in this study, reduced mitochondrial potential caused by MAHS expression could potentially indicate lower overall metabolic activity^87^. However, this is speculative, given that no prior studies of MAHS-influenced changes to mitochondrial potential have been performed. Mitochondria also contain ROS-neutralizing enzymes to counteract oxidative stress, including manganese superoxide dismutase, and the GPx4 isoform of glutathione peroxidase^88–93^, and expression of such enzymes may be upregulated in MAHS-expressing ASCs. Prior studies in HEp-2 cells also showed that transient MAHS expression was protective to hyperosmotic stress; a similar mechanism could be contributing here^60^.

### Differentiation

ASCs are partially characterized by their multilineage differentiation capacity: some common differentiation lineages for therapeutic applications include adipogenic and osteogenic cell types^94,95^. This allows for potential application of ASCs to treat a variety of conditions, including bone fractures, osteoarthritis and osteoporosis, spinal fusions, craniofacial and soft tissue defects, and cosmetic defects requiring enhanced adipogenic tissue volume^70,96–102^. Overall, the multilineage differentiation capacity in ASCs is a key contributor to their therapeutic potential. However, the presented studies in MAHS-expressing ASCs suggest that differentiation may be skewed in favor of osteogenic lineages. Neutral lipid droplet staining of MAHS-transgenic ASCs cultured in adipogenic media revealed a lower lipid count relative to ASCs expressing only AcGFP1, suggesting interference of the MAHS protein in ASC adipogenesis or lipogenesis (Fig. 5). Further, alkaline phosphatase staining of MAHS-transgenic ASCs cultured in osteogenic media demonstrated a higher level of alkaline phosphatase activity than ASCs expressing only AcGFP1 (Fig. 6).

While mechanism is not explored herein, one potentially compelling explanatory mechanism is hyperactivation of the AMPK/FOXO signaling pathway by MAHS through high basal AMPK concentrations. Expression of MAHS, an exogenous protein, may affect ATP generation in the mitochondria. Metabolic deficits can subsequently influence ASC differentiation processes through a number of regulators. Low glucose conditions activate both AMPK and FOXO to promote osteogenic over adipogenic differentiation^71^. Indeed, the restoration of adipogenic differentiation capacity in MAHS-ASCs following administration of excess glucose, a known AMPK inhibitor, to ASC cultures provides support for future investigation of AMPK as an effector of MAHS activity in ASCs (Figs. 7 and 8).

### Limitations and future work

Though MAHS expression in ASCs demonstrates significant potential for improved stress tolerance, several limiting factors must be considered. MAHS-transgenic ASCs are significantly less resistant to freezing, which is in contrast to the increased resistance of tardigrades to freezing (Figure S4)^60,78^. Additionally, MAHS-transgenic ASCs have only been analyzed in the context of constitutive expression; other expression modalities such as mRNA transfection may be more relevant to therapeutic applications^103^. Further, MAHS expression appears to push ASCs down an osteogenic lineage, which may be detrimental to therapeutic applications where non-osteogenic cell types are desired. However, bone and osteoarticular therapies may particularly benefit from targeted use of MAHS-expressing ASCs; this will be considered in future studies. Finally, there may be safety considerations associated with intrinsically disordered proteins, which should be assessed specifically for MAHS. As an example, amyloid-β peptide can form insoluble fibrillar protein aggregates, which have been linked to amyloidogenic-associated diseases, although specific mechanisms remain poorly understood^104–106^. Notably, there is no existing data describing the effect of MAHS expression on immunogenicity or its efficacy in vivo, although studies on other intrinsically disordered proteins indicate this may not be a concern^107,108^. Future studies will evaluate the viability and safety of MAHS expression in vivo*,* while mechanistic studies will be conducted on the molecular mechanisms downstream of MAHS that result in the functional changes observed.

### Supplementary Information

Supplementary Figures.

Supplementary Tables.

## Data Availability

The datasets generated and analyzed during the current study are available in supplementary materials. MATLAB analysis scripts are available at the lab website (https://timelab.engr.ucr.edu/matlab-tools), GitHub (https://github.com/UCRTIMELAB), and upon request.

## References

[1] Aly RM (2020). Current state of stem cell-based therapies: An overview. Stem Cell Investig..

[2] Stem Cell Therapy Market (By Product: Adult Stem Cells (ASCs), Human Embryonic Stem Cells (HESCs), Induced Pluripotent Stem Cells (iPSCs), Very Small Embryonic Like Stem Cells; By Therapy Type; By Application; By Technology; By End User)—Global Industry Analysis, Size, Share, Growth, Trends, Regional Outlook, and Forecast 2022–2030. https://www.precedenceresearch.com/stem-cell-therapy-market. Accessed 17 Aug 2023.

[3] Si Z (2019). Adipose-derived stem cells: Sources, potency, and implications for regenerative therapies. Biomed. Pharmacother..

[4] Tsuji W (2014). Adipose-derived stem cells: Implications in tissue regeneration. World J. Stem Cells.

[5] Yano F (2022). Effects of conditioned medium obtained from human adipose-derived stem cells on skin inflammation. Regen. Ther..

[6] Ceccarelli S, Pontecorvi P, Anastasiadou E, Napoli C, Marchese C (2020). Immunomodulatory effect of adipose-derived stem cells: The cutting edge of clinical application. Front. Cell Dev. Biol..

[7] Zuk PA (2001). Multilineage cells from human adipose tissue: Implications for cell-based therapies. Tissue Eng..

[8] Yubo M (2017). Clinical efficacy and safety of mesenchymal stem cell transplantation for osteoarthritis treatment: A meta-analysis. PLoS One.

[9] Zeng N, Chen H, Wu Y, Liu Z (2022). Adipose stem cell-based treatments for wound healing. Front. Cell Dev. Biol..

[10] Hassan WU, Greiser U, Wang W (2014). Role of adipose-derived stem cells in wound healing: Role of ASCs in wound healing. Wound Repair Regen..

[11] Wu H (2022). Engineered adipose-derived stem cells with IGF-1-modified mRNA ameliorates osteoarthritis development. Stem Cell Res. Ther..

[12] Freitag J (2019). Adipose-derived mesenchymal stem cell therapy in the treatment of knee osteoarthritis: A randomized controlled trial. Regen. Med..

[13] Qayyum AA (2017). Adipose-derived stromal cells for treatment of patients with chronic ischemic heart disease (mystromalcell trial): A randomized placebo-controlled study. Stem Cells Int..

[14] Chen C-F (2021). Treatment of knee osteoarthritis with intra-articular injection of allogeneic adipose-derived stem cells (ADSCs) ELIXCYTE^®^: A phase I/II, randomized, active-control, single-blind, multiple-center clinical trial. Stem Cell Res. Ther..

[15] Li X (2019). Harnessing the secretome of adipose-derived stem cells in the treatment of ischemic heart diseases. Stem Cell Res. Ther..

[16] Bora P, Majumdar AS (2017). Adipose tissue-derived stromal vascular fraction in regenerative medicine: A brief review on biology and translation. Stem Cell Res. Ther..

[17] Atesok K (2017). Stem cells in degenerative orthopaedic pathologies: Effects of aging on therapeutic potential. Knee Surg. Sports Traumatol. Arthrosc..

[18] Mazur P (1963). Kinetics of water loss from cells at subzero temperatures and the likelihood of intracellular freezing. J. Gen. Physiol..

[19] Gook DA, Edgar DH, Stern C (1999). Effect of cooling rate and dehydration regimen on the histological appearance of human ovarian cortex following cryopreservation in 1,2-propanediol. Hum. Reprod..

[20] Wang C, Xiao R, Cao Y-L, Yin H-Y (2017). Evaluation of human platelet lysate and dimethyl sulfoxide as cryoprotectants for the cryopreservation of human adipose-derived stem cells. Biochem. Biophys. Res. Commun..

[21] Yuan C (2014). Dimethyl sulfoxide damages mitochondrial integrity and membrane potential in cultured astrocytes. PLoS One.

[22] de Magalhães JP, Chainiaux F, Remacle J, Toussaint O (2002). Stress-induced premature senescence in BJ and hTERT-BJ1 human foreskin fibroblasts. FEBS Lett..

[23] Dludla PV (2018). A dose-dependent effect of dimethyl sulfoxide on lipid content, cell viability and oxidative stress in 3T3-L1 adipocytes. Toxicol. Rep..

[24] Crowley CA, Smith WPW, Seah KTM, Lim S-K, Khan WS (2021). Cryopreservation of human adipose tissues and adipose-derived stem cells with DMSO and/or trehalose: A systematic review. Cells.

[25] Halle P (2001). Uncontrolled-rate freezing and storage at −80 ℃, with only3.5-percent DMSO in cryoprotective solution for 109 autologous peripheral blood progenitor cell transplantations. Transfusion.

[26] Martín-Henao GA (2010). Adverse reactions during transfusion of thawed haematopoietic progenitor cells from apheresis are closely related to the number of granulocyte cells in the leukapheresis product: Adverse reactions during haematopoietic progenitor cell transfusion. Vox Sang..

[27] Lemarie C (2005). Clinical experience with the delivery of thawed and washed autologous blood cells, with an automated closed fluid management device: CytoMate. Transfusion.

[28] Mueller LP (2007). Neurotoxicity upon infusion of dimethylsulfoxide-cryopreserved peripheral blood stem cells in patients with and without pre-existing cerebral disease. Eur. J. Haematol..

[29] Zambelli A (1998). Clinical toxicity of cryopreserved circulating progenitor cells infusion. Anticancer Res..

[30] Davis JM, Rowley SD, Braine HG, Piantadosi S, Santos GW (1990). Clinical toxicity of cryopreserved bone marrow graft infusion. Blood.

[31] Amer MH, White LJ, Shakesheff KM (2015). The effect of injection using narrow-bore needles on mammalian cells: Administration and formulation considerations for cell therapies. J. Pharm. Pharmacol..

[32] Mamidi MK (2012). Impact of passing mesenchymal stem cells through smaller bore size needles for subsequent use in patients for clinical or cosmetic indications. J. Transl. Med..

[33] Tol M, Akar AR, Durdu S, Ayyildiz E, Ilhan O (2008). Comparison of different needle diameters and flow rates on bone marrow mononuclear stem cell viability: An ex vivo experimental study. Cytotherapy.

[34] Born C, Zhang Z, Al-Rubeai M, Thomas CR (1992). Estimation of disruption of animal cells by laminar shear stress. Biotechnol. Bioeng..

[35] Ko J, Park J, Kim J, Im G (2021). Characterization of adipose-derived stromal/stem cell spheroids versus single-cell suspension in cell survival and arrest of osteoarthritis progression. J. Biomed. Mater. Res. A.

[36] Kumar N, Saraber P, Ding Z, Kusumbe AP (2021). Diversity of vascular niches in bones and joints during homeostasis, ageing, and diseases. Front. Immunol..

[37] Filipowska J, Tomaszewski KA, Niedźwiedzki Ł, Walocha JA, Niedźwiedzki T (2017). The role of vasculature in bone development, regeneration and proper systemic functioning. Angiogenesis.

[38] Findlay DM (2007). Vascular pathology and osteoarthritis. Rheumatology.

[39] Zhang M (2001). Cardiomyocyte grafting for cardiac repair: Graft cell death and anti-death strategies. J. Mol. Cell. Cardiol..

[40] Müller-Ehmsen J (2002). Survival and development of neonatal rat cardiomyocytes transplanted into adult myocardium. J. Mol. Cell. Cardiol..

[41] Cree SJ, Smith RK, Dudhia J (2013). The effect of injection needle gauge size on the viability of equine mesenchymal stem cells. Equine Vet. J..

[42] Kibel A, Lukinac AM, Dambic V, Juric I, Selthofer-Relatic K (2020). Oxidative stress in ischemic heart disease. Oxid. Med. Cell. Longev..

[43] Bulkley GB (1987). Free radical-mediated reperfusion injury: A selective review. Br. J. Cancer Suppl..

[44] Mihai AD, Schröder M (2015). Glucose starvation and hypoxia, but not the saturated fatty acid palmitic acid or cholesterol, activate the unfolded protein response in 3T3-F442A and 3T3-L1 adipocytes. Adipocyte.

[45] Beegle JR (2016). Preclinical evaluation of mesenchymal stem cells overexpressing VEGF to treat critical limb ischemia. Mol. Ther. Methods Clin. Dev..

[46] Van Der Bogt KEA (2012). Molecular imaging of bone marrow mononuclear cell survival and homing in murine peripheral artery disease. JACC Cardiovasc. Imaging.

[47] Benoit E (2011). The role of amputation as an outcome measure in cellular therapy for critical limb ischemia: Implications for clinical trial design. J. Transl. Med..

[48] Aranguren XL, Verfaillie CM, Luttun A (2009). Emerging hurdles in stem cell therapy for peripheral vascular disease. J. Mol. Med..

[49] Ouma GO, Zafrir B, Mohler ER, Flugelman MY (2013). Therapeutic angiogenesis in critical limb ischemia. Angiology.

[50] Wang H (2017). A nano-in-micro system for enhanced stem cell therapy of ischemic diseases. ACS Cent. Sci..

[51] Eggenhofer E (2012). Mesenchymal stem cells are short-lived and do not migrate beyond the lungs after intravenous infusion. Front. Immunol..

[52] Banmeyer I (2004). Overexpression of human peroxiredoxin 5 in subcellular compartments of Chinese hamster ovary cells: Effects on cytotoxicity and DNA damage caused by peroxides. Free Radic. Biol. Med..

[53] Warner B, Papes R, Heile M, Spitz D, Wispe J (1993). Expression of human Mn SOD in Chinese hamster ovary cells confers protection from oxidant injury. Am. J. Physiol. Lung Cell. Mol. Physiol..

[54] Tamura T, McMicken HW, Smith CV, Hansen TN (1996). Overexpression Of human glutathione reductase in Chinese hamster ovary cells protects cells from oxidant injury ▴ 1474. Pediatr. Res..

[55] Elicker KS, Hutson LD (2007). Genome-wide analysis and expression profiling of the small heat shock proteins in zebrafish. Gene.

[56] Kayhan FE, Susleyici Duman B (2010). Heat shock protein genes in fish. Turk. J. Fish. Aquat. Sci..

[57] Anderson JM, Hand SC (2021). Transgenic expression of late embryogenesis abundant proteins improves tolerance to water stress in *Drosophila melanogaster*. J. Exp. Biol..

[58] Hesgrove C, Boothby TC (2020). The biology of tardigrade disordered proteins in extreme stress tolerance. Cell Commun. Signal..

[59] Fukuda Y, Miura Y, Mizohata E, Inoue T (2017). Structural insights into a secretory abundant heat-soluble protein from an anhydrobiotic tardigrade, *Ramazzottius varieornatus*. FEBS Lett..

[60] Tanaka S (2015). Novel mitochondria-targeted heat-soluble proteins identified in the anhydrobiotic tardigrade improve osmotic tolerance of human cells. PLoS One.

[61] Boothby TC, Pielak GJ (2017). Intrinsically disordered proteins and desiccation tolerance: Elucidating functional and mechanistic underpinnings of anhydrobiosis. BioEssays.

[62] Boothby TC (2017). Tardigrades use intrinsically disordered proteins to survive desiccation. Mol. Cell.

[63] Yamaguchi A (2012). Two novel heat-soluble protein families abundantly expressed in an anhydrobiotic tardigrade. PLoS One.

[64] Hashimoto T (2016). Extremotolerant tardigrade genome and improved radiotolerance of human cultured cells by tardigrade-unique protein. Nat. Commun..

[65] Ricci C (2021). The tardigrade damage suppressor protein modulates transcription factor and DNA repair genes in human cells treated with hydroxyl radicals and UV-C. Biology.

[66] Valente AJ, Maddalena LA, Robb EL, Moradi F, Stuart JA (2017). A simple ImageJ macro tool for analyzing mitochondrial network morphology in mammalian cell culture. Acta Histochem..

[67] Ruifrok AC, Johnston DA (2001). Quantification of histochemical staining by color deconvolution. Anal. Quant. Cytol. Histol..

[68] ColorDeconvolutionMatlab/ColorDeconvolutionDemo.m at master · jnkather/ColorDeconvolutionMatlab. *GitHub* https://github.com/jnkather/ColorDeconvolutionMatlab/blob/master/ColorDeconvolutionDemo.m. Accessed 15 Aug 2023.

[69] Pulido JS, Pulido CM, Bakri SJ, McCannel CA, Cameron JD (2007). The use of 31-gauge needles and syringes for intraocular injections. Eye.

[70] Romano IR (2023). Adipose-derived mesenchymal stromal cells: A tool for bone and cartilage repair. Biomedicines.

[71] Ahmadi A (2022). Recent advances on small molecules in osteogenic differentiation of stem cells and the underlying signaling pathways. Stem Cell Res. Ther..

[72] Furuki T (2020). Group 3 LEA protein model peptides suppress heat-induced lysozyme aggregation. Elucidation of the underlying mechanism using coarse-grained molecular simulations. J. Phys. Chem. B.

[73] Furuki T (2012). Effects of Group 3 LEA protein model peptides on desiccation-induced protein aggregation. Biochim. Biophys. Acta BBA Proteins Proteom..

[74] Furuki T, Sakurai M (2014). Group 3 LEA protein model peptides protect liposomes during desiccation. Biochim. Biophys. Acta BBA Biomembr..

[75] Shimizu T (2010). Desiccation-induced structuralization and glass formation of group 3 late embryogenesis abundant protein model peptides. Biochemistry.

[76] Zubillaga V, Alonso-Varona A, Fernandes SCM, Salaberria AM, Palomares T (2020). Adipose-derived mesenchymal stem cell chondrospheroids cultured in hypoxia and a 3D porous chitosan/chitin nanocrystal scaffold as a platform for cartilage tissue engineering. Int. J. Mol. Sci..

[77] Ernst P, Kim S, Yang Z, Liu XM, Zhou L (2023). Characterization of the far-red fluorescent probe MitoView 633 for dynamic mitochondrial membrane potential measurement. Front. Physiol..

[78] Richaud M (2020). Ultrastructural analysis of the dehydrated tardigrade *Hypsibius exemplaris* unveils an anhydrobiotic-specific architecture. Sci. Rep..

[79] Bartling B (2000). Shear stress-dependent expression of apoptosis-regulating genes in endothelial cells. Biochem. Biophys. Res. Commun..

[80] Leytin V (2004). Pathologic high shear stress induces apoptosis events in human platelets. Biochem. Biophys. Res. Commun..

[81] Hu Y, Hur SS, Lei L, Wang Y, Chien S (2017). Shear stress induces apoptosis via cytochrome C release from dynamic mitochondria in endothelial cells. FASEB J..

[82] Chiu JJ, Wung BS, Shyy JYJ, Hsieh HJ, Wang DL (1997). Reactive oxygen species are involved in shear stress-induced intercellular adhesion molecule-1 expression in endothelial cells. Arterioscler. Thromb. Vasc. Biol..

[83] Lv H (2015). Mechanism of regulation of stem cell differentiation by matrix stiffness. Stem Cell Res. Ther..

[84] Liu Y (2021). Stiffness regulates the morphology, adhesion, proliferation, and osteogenic differentiation of maxillary schneiderian sinus membrane-derived stem cells. Stem Cells Int..

[85] Nakayama Y, Mukai N, Kreitzer G, Patwari P, Yoshioka J (2022). Interaction of ARRDC4 with GLUT1 mediates metabolic stress in the ischemic heart. Circ. Res..

[86] Semenza GL (2000). Series introduction: Tissue ischemia: Pathophysiology and therapeutics. J. Clin. Invest..

[87] Garbern JC, Lee RT (2021). Mitochondria and metabolic transitions in cardiomyocytes: Lessons from development for stem cell-derived cardiomyocytes. Stem Cell Res. Ther..

[88] Miar A (2015). Manganese superoxide dismutase (SOD2/MnSOD)/catalase and SOD2/GPx1 ratios as biomarkers for tumor progression and metastasis in prostate, colon, and lung cancer. Free Radic. Biol. Med..

[89] Fridovich I (1995). Superoxide radical and superoxide dismutases. Annu. Rev. Biochem..

[90] Tao R (2010). Sirt3-mediated deacetylation of evolutionarily conserved lysine 122 regulates MnSOD activity in response to stress. Mol. Cell.

[91] Zhu Y (2012). Exploring the electrostatic repulsion model in the role of Sirt3 in directing MnSOD acetylation status and enzymatic activity. Free Radic. Biol. Med..

[92] Deisseroth A, Dounce AL (1970). Catalase: Physical and chemical properties, mechanism of catalysis, and physiological role. Physiol. Rev..

[93] Brigelius-Flohé R, Maiorino M (2013). Glutathione peroxidases. Biochim. Biophys. Acta BBA Gen. Subj..

[94] Grottkau BE, Lin Y (2013). Osteogenesis of adipose-derived stem cells. Bone Res..

[95] Yu G, Gimble JM, Bunnell BA (2011). Adipogenic differentiation of adipose-derived stem cells. Adipose-Derived Stem Cells.

[96] Surowiecka A, Strużyna J (2022). Adipose-derived stem cells for facial rejuvenation. J. Pers. Med..

[97] Tang H (2022). The therapeutic effect of adipose-derived stem cells on soft tissue injury after radiotherapy and their value for breast reconstruction. Stem Cell Res. Ther..

[98] Conese M (2020). The role of adipose-derived stem cells, dermal regenerative templates, and platelet-rich plasma in tissue engineering-based treatments of chronic skin wounds. Stem Cells Int..

[99] Issa MR, Naja AS, Bouji NZ, Sagherian BH (2022). The role of adipose-derived mesenchymal stem cells in knee osteoarthritis: A meta-analysis of randomized controlled trials. Ther. Adv. Musculoskelet. Dis..

[100] Ye X (2014). Adipose-derived stem cells alleviate osteoporosis by enchancing osteogenesis and inhibiting adipogenesis in a rabbit model. Cytotherapy.

[101] Hsu WK (2008). Stem cells from human fat as cellular delivery vehicles in an athymic rat posterolateral spine fusion model. J. Bone Jt. Surg. Am..

[102] Sheyn D (2011). Gene-modified adult stem cells regenerate vertebral bone defect in a rat model. Mol. Pharm..

[103] De Boeck J, Verfaillie C (2021). Doxycycline inducible overexpression systems: How to induce your gene of interest without inducing misinterpretations. Mol. Biol. Cell.

[104] Knowles TPJ, Vendruscolo M, Dobson CM (2014). The amyloid state and its association with protein misfolding diseases. Nat. Rev. Mol. Cell Biol..

[105] Ono K (2017). The oligomer hypothesis in α-synucleinopathy. Neurochem. Res..

[106] Sciacca MF (2020). Lipid-chaperone hypothesis: A common molecular mechanism of membrane disruption by intrinsically disordered proteins. ACS Chem. Neurosci..

[107] Esterly HJ (2020). Toxicity and immunogenicity of a tardigrade cytosolic abundant heat soluble protein in mice. Front. Pharmacol..

[108] Chang R (2022). Intrinsically disordered protein condensate-modified surface for mitigation of biofouling and foreign body response. J. Am. Chem. Soc..

