# Supplementary material for: The tardigrade-derived mitochondrial abundant heat soluble protein improves adipose-derived stem cell survival against representative stressors
Source: Sci Rep. 2024 May 23;14:11834. doi: 10.1038/s41598-024-62693-w (PMC11116449; doi:10.1038/s41598-024-62693-w)
Supplement: Supplementary file 1 — Supplementary Figures. [file 41598_2024_62693_MOESM1_ESM.docx]

**Supplemental Figures**


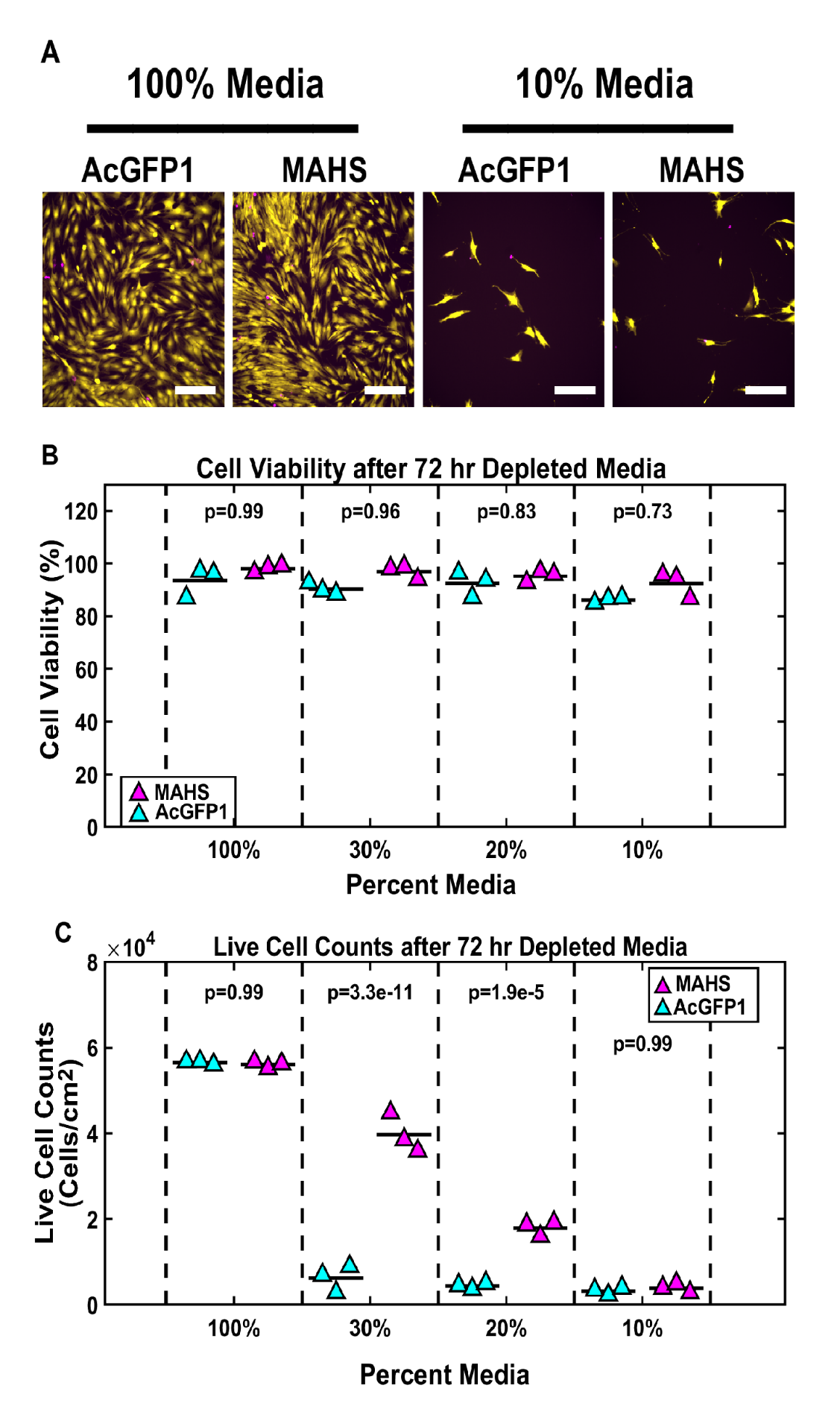


**Figure S1. Metabolic stress (cell viability). (A)** Maximum projections of live (Calcein-AM, yellow) and dead (EthD-III, magenta) AcGFP1- and MAHS-expressing ASC52telos following 72 hours of media depletion. Scalebars are 200 µm. **(B)** Quantification of cell viability in AcGFP1- and MAHS-expressing ASCs following a 72-hour media depletion (ANOVAN, Tukey’s HSD, *n = 3*). Denoted p-values indicate pairwise difference in cell survival between the genotypes at each specific media concentration. **(C)** Live cell count quantification in AcGFP1- and MAHS-expressing ASCs following a 72-hour media depletion (ANOVAN, Tukey’s HSD, *n = 3*). Denoted p-values indicate pairwise difference in cell survival between the genotypes at each specific media concentration.


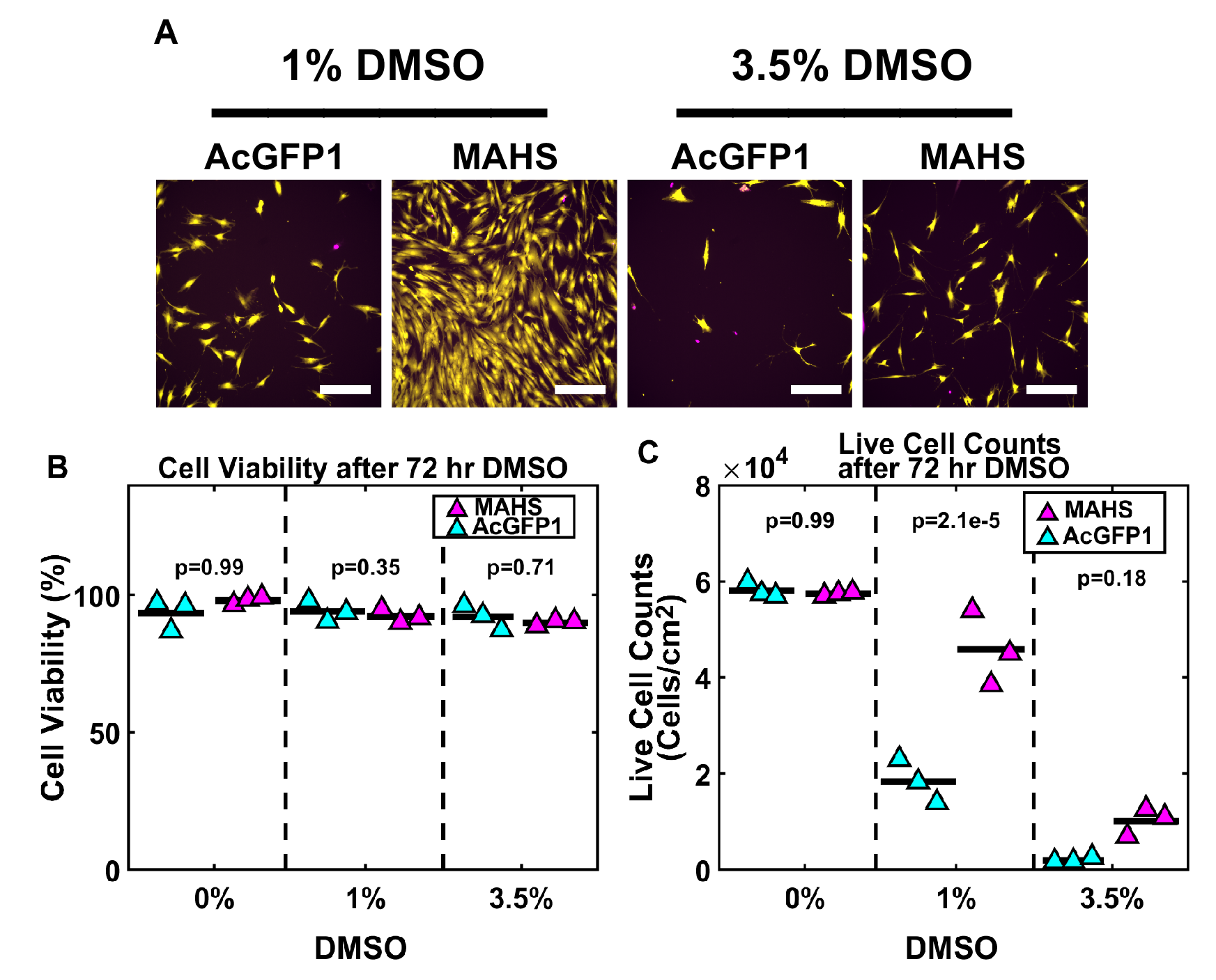


**Figure S2. DMSO stress (cell viability). (A)** Maximum projections of live (Calcein-AM, yellow) and dead (EthD-III, magenta) AcGFP1- and MAHS-expressing ASC52telos following 72 hours of DMSO treatment. Scalebars are 200 µm. **(B)** Quantification of cell viability in AcGFP1- and MAHS-expressing ASCs following a 72-hour DMSO treatment (ANOVAN, Tukey’s HSD, *n = 3*). Denoted p-values indicate pairwise difference in cell survival between the genotypes at each specific DMSO concentration. **(C)** Live cell count quantification in AcGFP1- and MAHS-expressing ASCs following a 72-hour DMSO treatment (ANOVAN, Tukey’s HSD, *n = 3*). Denoted p-values indicate pairwise difference in cell survival between the genotypes at each specific media concentration.


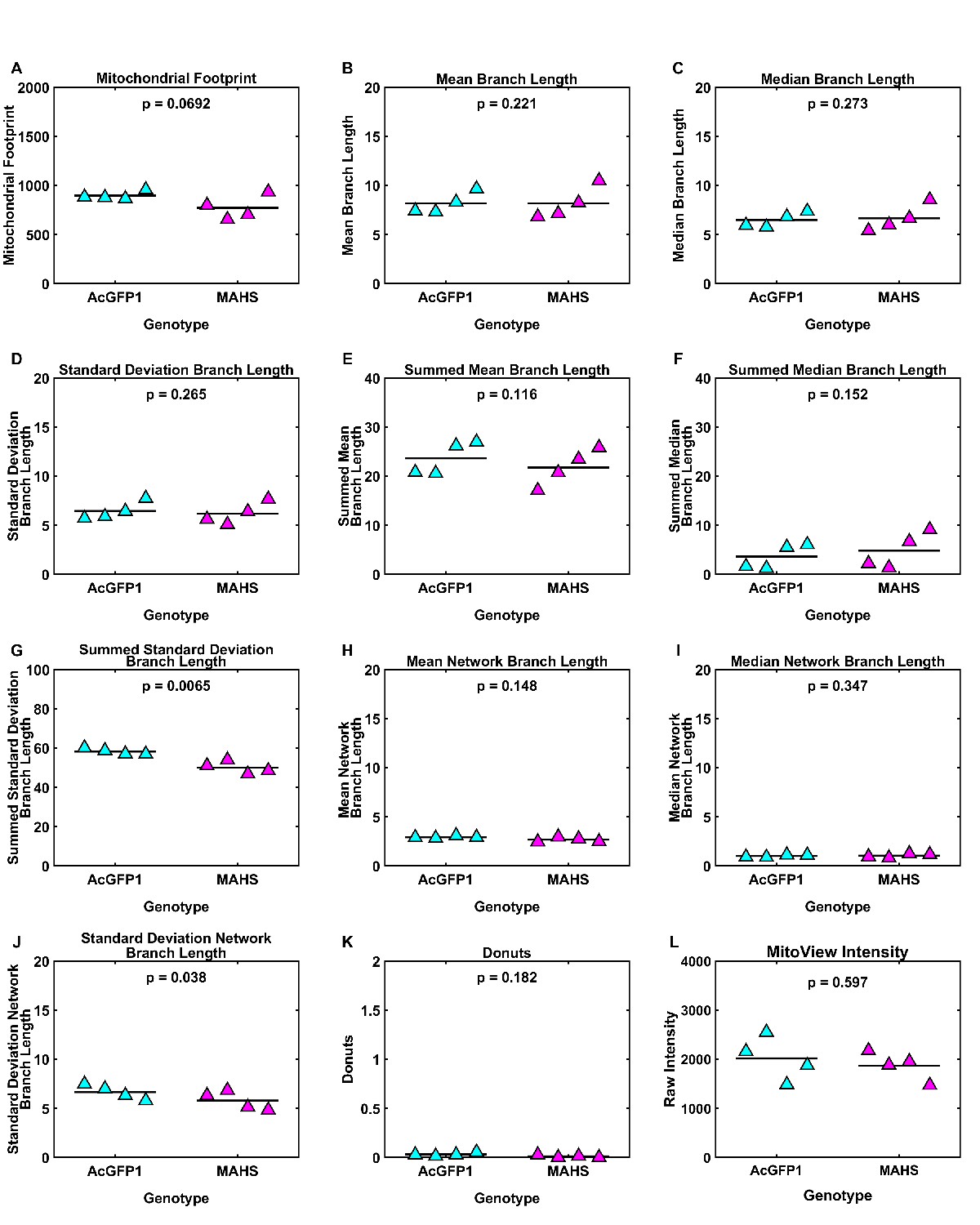


**Figure S3. Mitochondrial characterization.** Quantification of **(A)** Mitochondrial footprint (i.e., mitochondrial area), **(B)** mean mitochondrial branch length, **(C)** median mitochondrial branch length, **(D)** standard deviation of mitochondrial branch length, **(E)** summed mean of mitochondrial branch length, **(F)** summed median of mitochondrial branch length, **(G)** summed standard deviation of mitochondrial branch length, **(H)** mean mitochondrial network branch length, **(I)** median mitochondrial network branch length, **(J)** standard deviation of mitochondrial network branch length, **(K)** mitochondrial donuts, and **(L)** MitoView intensity in routinely cultured AcGFP1- and MAHS-transgenic ASCs (two-sample t-test, *n = 4)*. Denoted p-values indicate pairwise difference of each mitochondrial measurement between the genotypes.


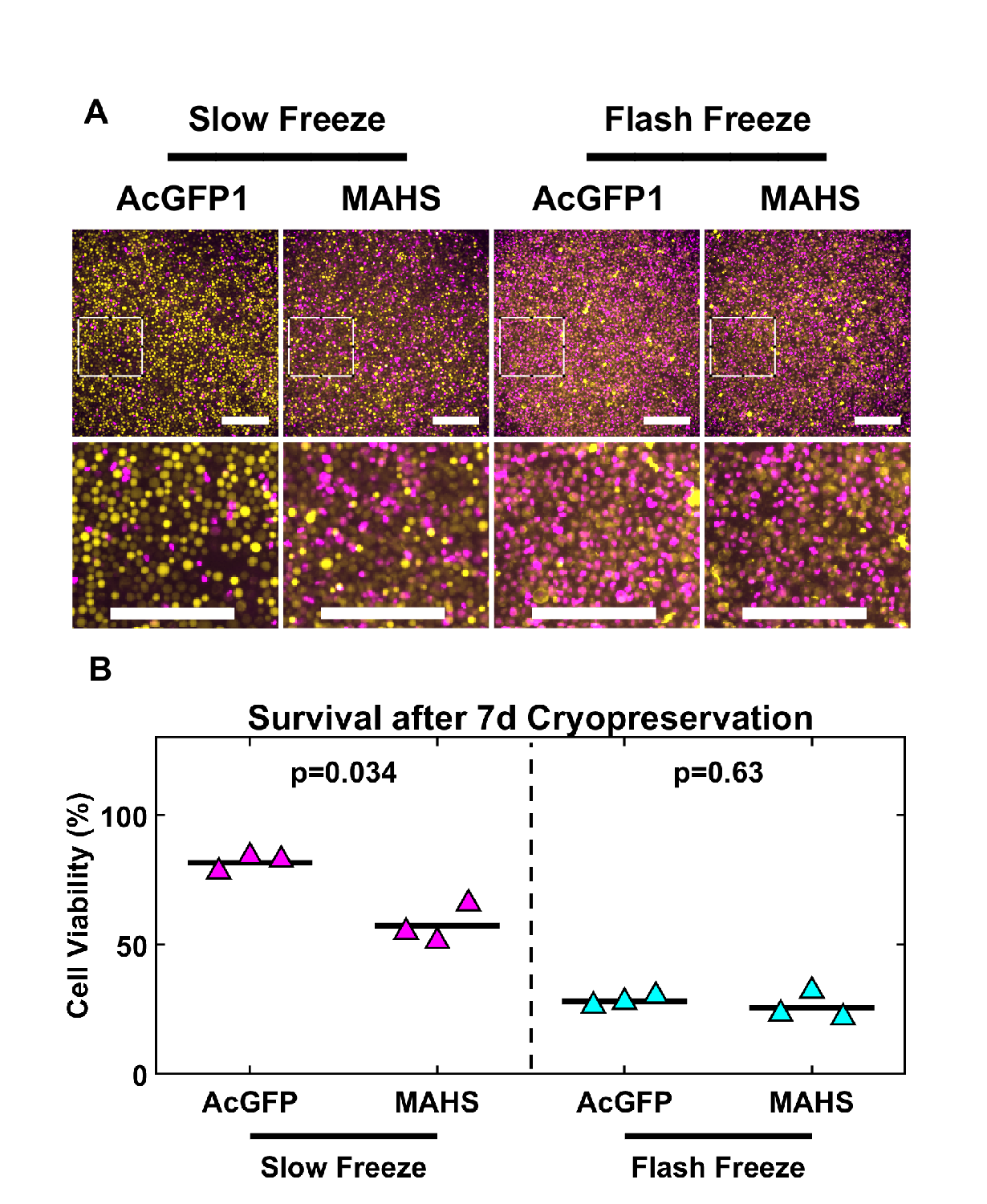


**Figure S4. Cryopreservation stress. (A)** Maximum projections of live (Calcein-AM, yellow) and dead (EthD-III, magenta) AcGFP1- and MAHS-transgenic ASC52telos 7 days after slow and flash freezing. Scalebars are 300 µm. (**B)** Quantification of cell survival in AcGFP1- and MAHS-transgenic ASCs 7 days after slow (cyan) and flash (magenta) freezing (two-sample t-test, *n = 3*). Denoted p-values indicate pairwise difference in cell survival between the genotypes for each freezing method.
